# High-Quality Deep Micro-Holes with Near-Zero Taper Efficiently Processed in CFRP Using a Picosecond Fiber Laser

**DOI:** 10.3390/polym18192325

**Published:** 2026-09-24

**Authors:** Kang Li, Jichao Yang, Chaowei Sun, Mengqi Suo, Mingyang Xin, Donghai Feng, Shian Zhang, Tianqing Jia, Zhenrong Sun, Hongxing Xu

**Affiliations:** 1 State Key Laboratory of Precision Spectroscopy, School of Physics and Electronic Science, East China Normal University, Shanghai 200241, China; 2 Basic Science Center Project of Light Manufacturing Science and Engineering, Institute of Laser Manufacturing, Henan Academy of Sciences, Zhengzhou 450046, China; 3 Micius Laboratory, Henan Academy of Sciences, Zhengzhou 450046, China

**Keywords:** deep micro-holes, carbon fiber reinforced polymer (CFRP), picosecond fiber laser, two-step processing strategy, spiral scanning machining head

## Abstract

Carbon fiber reinforced polymer (CFRP) is extensively utilized in aerospace, transportation, and particularly in the rapidly evolving low-altitude economy sector represented by unmanned aerial vehicles and electric vertical take-off and landing aircraft. However, laser drilling of CFRP presents significant challenges, especially in processing deep micro-holes, due to pronounced thermal effects and hole taper. In this study, a picosecond fiber laser beam was directed into a spiral scanning machining head to establish a precision micro-hole machining system. A two-step machining strategy assisted by high-pressure compressed air was proposed: the first step employs full spiral scanning for efficient through-hole drilling, while the second step utilizes half spiral scanning to form near-zero taper circular holes. The through-hole generated in the first step facilitates the passage of high-pressure assist gas, effectively removing ablation debris and providing cooling to the inner wall. Circular holes with a diameter of 400 μm and an aspect ratio of 5:1 were successfully fabricated on CFRP with high efficiency. Both the hole entrance/exit and the inner wall exhibited excellent quality: the heat-affected zone width at the entrance was 8.1 μm, with a taper as low as 0.0045. The inner wall was extremely smooth, featuring an average roughness of 0.379 μm. The fibers and resin remained tightly bonded, and the material structure at the fiber ends was well preserved. The system demonstrated high processing efficiency and excellent consistency in fabricating deep micro-hole arrays, indicating strong potential for industrial applications.

## 1. Introduction

Carbon fiber reinforced polymers (CFRPs) exhibit outstanding characteristics such as low density, high specific strength, corrosion resistance, and fatigue resistance [1,2,3,4], which have led to their wide application in industries including aerospace, automotive manufacturing, transportation, and medical devices [5,6]. With the rapid development of the global low-altitude economy, CFRP is playing an increasingly critical role in the lightweight design of unmanned aerial vehicles (UAVs) and electric vertical take-off and landing (eVTOL) aircraft [7].

In practical applications, CFRP components often require joining and assembly, which inevitably involves essential processing such as drilling and cutting. Current primary processing methods include mechanical machining, ultrasonic vibration-assisted machining (UVAM), electrical discharge machining (EDM), and laser machining. Due to the significant differences in strength, toughness, and anisotropy between carbon fibers and the epoxy resin matrix, mechanical machining induces various defects such as delamination, burrs, fiber pull-out, spalling, and matrix cracking, severely deteriorating the mechanical properties of CFRP [8,9]. Moreover, these processes are often accompanied by significant tool wear, which further exacerbates defects like delamination and burrs [10]. UVAM exhibits relatively low processing efficiency and remains constrained by the inherent limitations of mechanical machining [11,12]. EDM generates substantial thermal effects while also demonstrating relatively low machining accuracy and efficiency [13,14].

Laser machining offers distinct advantages, including non-contact operation, absence of tool wear, and ease of control [15,16,17,18]. However, this technique still faces several primary challenges: a significant heat-affected zone (HAZ) at the hole entrance, pronounced hole taper, and severe defects on the inner wall surface [19,20]. To address these challenges and enhance both drilling quality and efficiency, various methods and strategies, such as ultrafast laser machining and spiral drilling processes, have been developed [21,22,23].

Femtosecond laser processing offers the advantages of minimal thermal effects and high precision, enabling the high-quality fabrication of holes with diameters larger than 1 mm or shallow micro-holes on CFRP plates [24,25]. However, there have been few reports on the drilling of high-quality deep micro-holes (diameter < 500 µm, aspect ratio > 5:1). Such deep micro-holes in CFRP hold broad application prospects in fields such as fiber-optic sensors, medical devices, UAVs, and the assembly of miniature precision components [26,27]. However, drilling deep micro-holes faces multiple challenges, including difficulties in removing ablated materials, adhesion of debris to the hole walls, strong plasma shielding effects, and severe heat accumulation. These factors collectively make it difficult to achieve high-quality, near-zero taper deep micro-holes.

Compared with femtosecond lasers, picosecond fiber lasers exhibit notable advantages such as higher power, excellent stability, and ease of integration, demonstrating broad prospects for industrial applications [28,29,30]. However, the longer pulse width of picosecond lasers leads to pronounced thermal effects during processing. Therefore, utilizing picosecond fiber lasers for deep micro-hole drilling represents a research topic that is not only highly promising but also considerably more challenging.

In this study, a picosecond fiber laser beam was delivered into a spiral scanning machining head (SSMH), which was integrated with a five-axis machine tool to establish a precision micro-hole processing system. By adjusting the taper and aperture parameters of the SSMH, circular holes with specified diameters and near-zero taper were successfully fabricated. A two-step processing strategy assisted by high-pressure gas was proposed, which has been successfully employed to mitigate taper and suppress HAZ in micro-holes drilled in superalloys and ceramic matrix composites [31,32,33,34]. In the first step, full spiral scanning was employed to achieve efficient penetration. The effects of pulse repetition frequency, starting position of focal point descent, and SSMH taper parameters on processing efficiency, entrance/exit diameters, and HAZ width were investigated, and the processing parameters were studied accordingly. In the second step, half spiral scanning was utilized to produce near-zero taper circular holes matching the target aperture by fine-tuning the SSMH taper and aperture parameters. The tapered through-hole generated in the first step facilitated the flow of high-pressure auxiliary gas, which effectively expelled ablation debris, reduced plasma shielding, cooled the inner wall of the hole, and thereby significantly lowered thermal effects during the second-step machining. As a result, both processing efficiency and quality were simultaneously improved.

On a 2 mm-thick CFRP plate, near-zero taper circular holes with a diameter of 400 μm were processed, achieving an aspect ratio of 5:1. The hole quality was excellent: both the entrance and exit exhibited high roundness of 96% and an extremely low taper of 0.0045. The HAZ width was also very small, measuring 8.1 μm. The inner wall of the holes was free of molten debris and displayed a smooth, flat surface with an average roughness of 0.379 μm. The bonding between the fibers and the resin remained well, with an average fiber protrusion above the resin in adjacent gaps of 0.32 μm. The crystalline structure of the fibers along the hole wall remained intact, showing no significant lattice defects. The two-step processing strategy demonstrated high efficiency, with a single micro-hole completed in only 180 seconds. Moreover, the system exhibited good stability: a 10 × 10 array of deep micro-holes was continuously fabricated on a test plate, showing high consistency in aperture of 400 ± 25 μm and HAZ widths below 10 μm.

## 2. Materials and Methods

### 2.1. Materials

Commercial CFRP plates, consisting of T300 carbon fibers and YB01 epoxy resin matrix, were used as the experimental samples, which were provided by Jiangsu Boshi (Zhenjiang, China). As depicted in Figure 1, the material was prepared from 3K plain-weave carbon fiber prepregs using a compression molding technique [23]. Both the top and bottom surface layers consist of woven carbon fiber fabric with a single-layer thickness of 230 μm. The internal 11 plies are arranged in a unidirectional ply structure, with adjacent plies oriented alternately at 0° and 90°. The thickness of each ply varied from 100 to 160 μm, with an average thickness of approximately 140 μm. The total thickness of the sample is 2 mm. The diameter of a single carbon fiber is approximately 7 μm, and the fiber volume fraction is 60%. The physical properties of the constituent materials are listed in Table 1 [6,35].

### 2.2. Experimental Setup

The laser used in the experiments was a diode-pumped ytterbium-doped picosecond fiber laser (YLPP-100-1-100-R, IPG Photonics, Marlborough, MA, USA), with its specific parameters detailed in Table 2. The maximum repetition frequency of the laser is 1000 kHz, which can be reduced by a factor of 1/n. In this study, a low laser frequency was adopted, mainly to reduce the thermal accumulation effect during laser drilling. The linearly polarized laser was first converted into circularly polarized light using a quarter-wave plate. It was then directed by mirrors into an SSMH (KH30A, Shangtai Optoelectronics, Xi’an, China) and subsequently focused by an f = 100 mm lens, yielding a focused spot diameter of 25 μm.

During the drilling process, compressed air at a pressure of up to 0.65 MPa was delivered vertically downward to the laser focal region through a conical nozzle to remove ablation debris and cool the processing zone. The inner diameter of the exit of the conical nozzle was 3.0 mm, and the distance between the nozzle outlet and the specimen surface was 10.4 mm. The specimen was clamped in a vise and mounted on a cradle-type rotary stage. The five-axis platform was supplied by AEROTECH (Pittsburgh, PA, USA). The travel ranges of the X-, Y-, and Z-axes were 600 mm, 300 mm, and 300 mm, respectively, with an accuracy of ±0.4 μm for each axis. The tilting rotary stage was model APR 200 with an accuracy of 1 arc sec, while the rotary stage was model ADRS 150 with an accuracy of 3 arc sec. The processing was monitored in real-time by an off-axis illumination imaging system, as shown in Figure 2a.

The schematic of the optical path of the SSMH is illustrated in Figure 2c, which mainly consists of a deflecting wedge couple, a displacement wedge couple, and a focusing lens. When an incident laser passes through a single optical wedge, its deflection angle is (n − 1)α, where α is the wedge angle and n is the refractive index of the wedge. When the beam passes through the deflection wedge couple, the total deflection angle δ varies with the phase difference φ between the principal sections of the two wedges, known as the Wedge Phase Deviation Angle (WPDA): δ = 2 (n − 1)α cos φ. This deflection angle δ, or equivalently the WPDA φ, primarily determines the scanning radius of the laser focal spot.

The displacement wedge couple consists of two parallel optical wedges placed in opposite orientations. After passing through this optical assembly, the laser beam undergoes lateral displacement, the magnitude of which varies with the distance L between the two wedges, referred to as the Wedge Displacement (WD). The WD primarily influences the angle of incidence (AOI, denoted as θ) of the laser beam, which in turn determines the taper of the machined hole. The incident angle is defined as positive when the laser beam directs from the hole edge toward the central axis, and negative when it directs from the central axis toward the hole edge. Table 3 lists the AOI values corresponding to different WD settings. In practical processing, the taper is also influenced by other parameters, including laser pulse energy, pulse width, repetition frequency, as well as scanning speed of SSMH and assisted air pressure. Finally, the laser beam is focused onto the focal plane with a specific AOI.

When the relative positions of the two wedge couples are fixed and they rotate synchronously at the same angular velocity, the laser beam will form a circular scanning trajectory on the focal plane. When the two wedge couples rotate at specific speeds and the WPDA of the deflection couple varies cyclically within a set range, a fully spiral or a half spiral scanning path with a specific radius can be achieved, as illustrated in Figure 2(d1–d4). The displacement wedge couple deflects the laser beam, causing the beam trajectory on the focal plane to be non-uniformly distributed, but rather gradually concentrated toward the outer ring, as shown in Figure 2(e1–e4). A larger WD results in a more pronounced concentration of the trajectory toward the outer ring.

### 2.3. Strategy and Method: Two-Step Processing

Compared to the epoxy resin matrix, carbon fibers exhibit higher absorptivity under 1030 nm laser irradiation [36,37], with a vaporization temperature exceeding the thermal decomposition temperature of the resin by approximately 3200 K. Furthermore, the axial thermal conductivity of carbon fibers is approximately ten times greater than their radial value, causing heat to preferentially propagate along the fiber axis. Under laser irradiation, the resin reaches its thermal decomposition temperature first and is subsequently removed, resulting in a large HAZ, particularly in the direction perpendicular to the carbon fibers [16,38]. Consequently, significant challenges arise in the drilling of deep micro-holes, including inefficient ejection of ablated material and the adhesion of debris to the hole walls.

To achieve high-quality and high-efficiency drilling of deep micro-holes using a picosecond laser, this study proposes a two-step processing strategy, as illustrated in Figure 3. The first step employs a full spiral scanning method, aiming to rapidly penetrate and form a through-hole while minimizing the formation of HAZ. During this step, parameters such as the pulse repetition frequency, WD of the SSMH, and the starting position of the laser focus were studied with the objectives of minimizing the perforation time, reducing the HAZ width, and maximizing the exit hole diameter. The spiral scanning path diameter was set to 390 μm.

In the second processing step, a half-spiral scanning method was employed. Here, “half-spiral” is used in contrast to a full spiral, and generally refers to a processing pattern in which the central portion is not completely filled by the spiral path. The laser incidence angle was adjusted by varying the WD of SSMH, ultimately producing a near-zero taper circular hole that conforms to the designed specifications. The outer and inner diameters of the scanning path were 400 μm and 225 μm, respectively. During the process, auxiliary high-pressure gas went through the hole formed in the first step, efficiently expelling the majority of ablated materials from the exit. This significantly reduces debris accumulation at the entrance, narrows the HAZ around the entrance region, and improves the circularity of the hole orifice. The efficiency of ablative material removal via auxiliary gas flow from the exit far surpasses that of back-sputtering, thereby substantially mitigating the plasma shielding effect caused by ablation ejecta and reducing debris deposition on the inner wall. Furthermore, the high-pressure gas flow provides effective cooling along the inner wall. This alleviates the temperature rise inside the hole induced by the strong absorption of subsequent laser pulses by the ejecta, as well as mitigates crack formation and delamination caused by shock waves. Consequently, the processing quality of both the hole entrance/exit and the inner wall is significantly improved.

### 2.4. Post-Processing, Measurements and Characterizations

Following laser processing, the samples were ultrasonically cleaned in anhydrous ethanol. Subsequently, they were sectioned parallel to the hole axis using a precision cutting machine. The sectioned samples were then mounted in resin with a curing agent, ensuring that the hole axis remained parallel to the base of the mounting mold. The mounted cross-sections underwent coarse and fine grinding/polishing. Lastly, an ultrasonic cleaning in anhydrous ethanol was performed.

The surface and cross-sectional morphologies of the samples were characterized using a scanning electron microscope (SEM, S-4800, Hitachi, Tokyo, Japan). The three-dimensional topography of the inner wall was examined using a confocal optical microscope (COM, VK-X3000, Keyence, Osaka, Japan) with a 50× objective. The lateral (X–Y) and axial (Z) resolutions are 0.25 μm and 1.7 nm, respectively. Raman spectra were measured using a Raman spectrometer (inVia Qontor, Renishaw, Gloucestershire, UK) equipped with a 532 nm excitation laser to characterize structural changes in the material.

As illustrated in Figure 4a, the width of HAZ is defined as follows:

(1) $$W_{HAZ}\;=\;\frac{S_{2}-S_{1}}{2\pi \sqrt{\left(S_{1}+S_{2}\right)/2\pi }},$$

where *S*_2_ represents the area enclosed by the outer boundary of the HAZ and *S*_1_ denotes the area of the drilled hole.

Due to the laser Gaussian distribution and beam shielding by the hole wall, the entrance diameter of a laser-drilled hole is typically larger than that of the exit, resulting in a certain taper. The taper *T* is defined as follows [39]:

(2) $$T\;=\;\frac{D_{entrance}\;-{\;D}_{exit}}{2h},$$

where *D_entrance_* and *D_exit_* represent the entrance and exit diameters, respectively, and h is the sample thickness.

To enhance the accuracy of the results and minimize errors, four sets of experiments were conducted under each condition.

## 3. Results and Discussion

During the two-step processing, the local geometry of the sample transitions from a flat plate into micro-holes with varying diameters and taper. The processing objectives also differ significantly—from achieving rapid perforation to finally machining through-holes with a specified diameter (400 μm) and near-zero taper. Accordingly, the laser-material interaction mechanisms and the process parameters vary distinctly between the different processing steps. This section systematically investigates the influence of WD on hole taper and diameter. By systematically varying the pulse repetition frequency, focus offset, and assisted gas pressure, the effects of these parameters on the entrance/exit diameter, HAZ width, as well as the inner-wall morphology, surface roughness, and lattice structural characteristics of the carbon fibers were studied in detail.

### 3.1. Step 1: Efficient Perforation via Full Spiral Scanning

The primary objective of Step 1 is to rapidly process a through-hole, enabling the smooth passage of high-pressure assisted gas to achieve efficient removal of ablation debris from the deep micro-hole and to cool the hole itself. During this initial perforation stage, the target width of HAZ at the entrance is controlled within 10 μm. A full spiral scanning strategy is employed.

In the following, the influence of pulse repetition frequency, focus offset, and WD of the SSMH on perforation efficiency and HAZ width is systematically investigated, and the underlying mechanisms are discussed.

#### 3.1.1. Pulse Repetition Frequency on Piercing Efficiency and Hole Morphology

During the Step 1, the picosecond fiber laser operates at a maximum repetition frequency of 20 kHz, corresponding to a relatively short inter-pulse interval of 50 µs. When subsequent pulses arrive, the focus area remains at an elevated temperature, and the ablated ejecta do not fully escape from the focal region. Therefore, the repetition frequency significantly influences ablation efficiency, thermal accumulation effects, and plasma shielding. The experimental parameters were set as follows: a laser pulse energy of 90 µJ, with the repetition frequency varied over the range of 3–20 kHz. The WD of the SSMH was fixed at 30 mm.

In the first step, the laser focus was moved at a constant speed from the top to the bottom surface of the sample. The feed rate *v* was set to be h/*v* = 1.1 t_p_, where h is the sample thickness, t_p_ is the piercing time measured at each pulse repetition frequency. The factor of 1.1 was introduced to provide a 10% time margin, ensuring that the sample was completely penetrated. When the pulse repetition frequency was adjusted to 3, 5, 10, 15, and 20 kHz, the corresponding feed rates were set to 0.0055, 0.01, 0.025, 0.04, and 0.055 mm/s, respectively. The results in Figure 5a show that a higher pulse repetition frequency enables more efficient perforation, requiring a lower cumulative pulse count. As the repetition frequency decreased from 10 kHz to 3 kHz, the cumulative pulse number required for perforation increased sharply from 670 k to 970.5 k. These results indicate that the longer pulse interval (333 µs) led to a more pronounced drop in material temperature before the arrival of the subsequent pulse, accompanied by a reduction in the pulse overlap ratio. The two factors acted together, resulting in a decline in ablation efficiency. However, when the pulse repetition frequency was reduced from 20 kHz to 10 kHz, the change in the required cumulative pulse number was relatively small, increasing only from 620 k to 670 k. This suggests that the decrease in ablation efficiency and the mitigation of the plasma shielding effect exerted nearly equal and opposing influences on perforation efficiency.

Figure 6 shows the entrance morphology after laser perforation at different repetition frequencies. For a repetition frequency of 20 kHz, the entrance exhibits the typical asymmetric ablation morphology for CFRP. The laser energy absorbed by the CFRP propagates along the carbon fibers. Due to the significantly higher axial thermal conductivity compared to the radial conductivity of the carbon fibers, and the much lower thermal decomposition temperature of the resin than the vaporization temperature of the carbon fibers, asymmetric ablation of the resin occurs [24]. This process leads to the separation and detachment of carbon fibers, resulting in pronounced fiber pull-out accompanied by swelling at the fiber ends. As shown in Figure 6e,f, the ablation zone exhibits an elliptical shape, while the hole entrance remains circular, with an average HAZ width of 51.4 µm.

Figure 5 and Figure 6 demonstrate that when the repetition frequency is reduced to 15 kHz and 10 kHz, HAZ width decreases to 22.1 μm and 10.4 μm, respectively. This indicates that lowering the repetition frequency has a significant effect on reducing the HAZ size [40]. This phenomenon is attributed to the increased time interval between pulses at lower repetition frequencies, which allows the heat generated by laser ablation to dissipate more effectively through convection by the high-pressure assist gas, as well as through heat conduction and radiation, thereby reducing the heat accumulation [41,42,43]. However, when the repetition frequency was further reduced to 3 kHz, the HAZ width increased to 11.6 μm, accompanied by local chipping. This is because an excessively low repetition frequency leads to a prolonged interval between pulses, resulting in a substantial temperature drop of the material at the focal point, which significantly reduces the perforation efficiency. As shown in Figure 5a, the perforation time is extended in this case, and the cumulative number of pulses increases markedly, ultimately leading to an increase in the HAZ width.

At a repetition frequency of 10 kHz, the perforation time was 67 s, representing a reduction of 60.0% and 79.3% compared to the cases at 5 kHz and 3 kHz, respectively. The average HAZ width was 10.4 μm, which is 79.8% smaller than that of the hole processed with a 20 kHz repetition frequency. Considering both perforation efficiency and thermal effects, a repetition frequency of 10 kHz was selected for Step 1.

#### 3.1.2. Effects of WD on Piercing Efficiency and Hole Morphology

WD of SSMH is a key parameter that controls the propagation of the laser beam along the optical axis, directly determining the hole taper and aperture size [44,45,46]. Figure 7 and Figure 8 show the variations in the morphology, diameter, HAZ width, and perforation time as WD changes from 10 mm to 50 mm in increments of 10 mm. The laser focus was moved from the top to the bottom surface at a constant speed of 0.02 mm/s.

Figure 7 and Figure 8 show that all of the entrances exhibited regular circular shapes. The exit diameter gradually expanded, and the perforation time increased from 55 s to 86 s as WD increased to 50 mm. Due to the low pulse repetition frequency of 10 kHz, the average HAZ width at the entrances remained below 15 µm. The minimum HAZ width was observed at a WD of 30 mm, measuring only 10.4 µm. No distinct HAZ was observed at any of the exits.

Figure 9 shows the cross-sectional morphology of the holes processed with different WD. Due to the low pulse repetition frequency of 10 kHz, the heat accumulation effect was minimal, and no significant melting or ablation of the matrix resin was observed surrounding the hole wall caused by heat transfer along the fiber direction. At a WD of 10 mm, pronounced cracking appeared on the inner wall near the entrance. The exit diameter was noticeably reduced, and occasional fiber detachment was observed. As shown in Table 3, AOI for WD = 10 mm was a small positive angle. During the piercing process, the hole walls caused obstruction and scattering of the laser beam [47,48], resulting in a significant reduction in exit diameter and the formation of a large taper. As shown in Figure 9a, over-processing of the entrance and the adjacent inner walls induced extensive cracking and increased the HAZ size. Since the laser energy was highly concentrated in the central region of the hole, the perforation efficiency was very high.

As WD increased from 10 mm to 30 mm, the inner wall became much smoother. Defects such as fiber detachment and cracking were markedly reduced, with only occasional debris deposition. This improvement can be attributed to the formation of a slight inverted conical structure in the laser scanning trajectory when the WD was set to 30 mm. This geometric configuration reduces the obstruction and scattering of the laser beam by the hole wall, thereby preventing excessive processing of the entrance wall and resulting in a noticeable expansion of the exit diameter. The larger hole diameter facilitates more complete expansion of the ablation products and enhances their expulsion from the hole by the assist gas. This not only significantly mitigates thermal effects but also improves the quality of the inner wall surface.

However, as WD was further increased to 40 mm and 50 mm, a significant expansion section appeared in the middle part of the hole. The inner walls exhibited deep grooves accompanied by distinct cracks. The exit section became extremely rough, with many stripes and cracks on the surface. It is evident that the thermal effects increased dramatically during the drilling process as WD = 50 mm.

To investigate the reasons for the sharp decline in hole wall quality accompanied by bulging in the middle section when WD exceeded 40 mm, the cross-sectional morphology of blind holes drilled for different durations under a constant feed rate of 0.02 mm/s was further observed and analyzed. Figure 10 demonstrates significant differences in the drilling process when WD was 30 mm and 50 mm, respectively. Although the laser scanning path itself exhibited a slight inverted conical distribution at WD = 30 mm, images Figure 10(a1–a3) show that the hole wall maintained a positive taper profile throughout the drilling process, while the central micro-pointed cone gradually elongated. This phenomenon is attributed to the large-angle reflection of the laser beam by the hole wall. The positively tapered hole geometry facilitates the effective ejection of ablation products through reverse jetting from the entrance, thereby resulting in a regular inner wall.

Figure 10c,d present a statistical analysis of variations in drilling depth and drilling rate across different stages, revealing that laser drilling efficiency changes significantly with increasing depth. The drilling rate at the exit was only 9.6 µm/s, less than 1/6 of that at the entrance. This phenomenon can be primarily attributed to two factors: first, the attenuation of laser intensity due to occlusion and scattering by the hole wall, which becomes more pronounced with increasing depth; second, during micro-hole drilling, the efficiency of ablative product expulsion gradually decreases and the plasma shielding effect intensifies as the depth increases.

When WD was increased to 50 mm, AOI reached a large value of −3.37°. As shown in Figure 2(e2), the laser focus deflected toward the outer edge, and the scanning trajectory generated a quasi-hollow annular structure. When the processing time reached 20 s and the focal point was lowered by 0.4 mm, the laser processed a blind hole with a depth of 0.98 mm in an annular cutting mode, leaving a central cone. As shown in Figure 10(b1), the kerf in the lower half of the blind hole was extremely narrow, with a width of only 30–50 μm. This narrow kerf significantly reduced the expulsion efficiency of the ablated material and enhanced the plasma shielding effect, thereby decreasing the drilling rate. Consequently, when the processing time was extended from 20 s to 40 s, the depth of the blind hole increased by only 200 μm, as shown in Figure 10b,c. The average drilling rate during this stage was extremely low, measuring only 10 μm/s. This indicates that a substantial amount of laser energy was deposited in the middle section of the hole, leading to the widening of the kerf in the lower half of the blind hole and the onset of irregular bulging on the hole wall, as shown in Figure 10(b2).

When the processing time was increased to 60 s, the blind hole reached a depth of 1.6 mm, and its bulging section was essentially formed. During this process, the ejecta could only escape through reverse ejection along the kerf walls, causing the plasma plume to continuously erode the hole walls and form multiple prominent grooves on both the inner wall surface and the central cone surface [40,47]. Intense thermal effects led to the formation of numerous cracks. Due to large-angle reflection and scattering from the inner wall, which caused the beam to deflect toward the center along with attenuation of laser intensity, the diameter of the middle-to-lower section gradually contracted, resulting in an asymmetric spindle-shaped morphology, as shown in Figure 10(b3). The narrow kerf formed by annular cutting at the exit section, together with the reverse ejection of ablated material initiated from deeper regions, led to low expulsion efficiency. A substantial amount of resin adhered to the inner wall, resulting in a very rough surface. Figure 10(b3) shows that the central cone fractured, enabling clear observation of the inner wall morphology of the blind hole.

Based on a comprehensive consideration of the HAZ width, exit diameter, processing efficiency, and inner wall quality, a WD of 30 mm was selected for further processing in Step 1.

#### 3.1.3. Effects of the Starting Position of Laser Focus on Piercing Efficiency and Hole Morphology

Figure 10 shows that the processing efficiency at the entrance is significantly higher than that at the exit. Therefore, adjusting the starting position *Z_start_* of the laser focus to deposit more laser energy in the middle and lower regions of the hole is beneficial for improving drilling efficiency [42,49]. In the experiment, the starting position *Z_start_* was varied from 0 mm (on the top surface) to 1.6 mm (1.6 mm below the top surface) in increments of 0.4 mm, while the termination position was fixed at the bottom surface of the sample (i.e., *Z_end_* = 2 mm). To ensure a consistent processing time of 100 s for different *Z_start_* values, the feed rate *v* was adjusted to satisfy the relation (*Z_end_* − *Z_start_*)/*v* = 100 s. The WD was fixed at 30 mm.

Figure 11 shows the variations in the entrance diameter, exit diameter, HAZ width, and piercing time with respect to *Z_start_*. As *Z_start_* increases, the exit diameter gradually increases, reaching a maximum value of 265 μm at *Z_start_* = 0.8 mm. Upon further lowering of *Z_start_*, the exit diameter gradually decreases. Figure 11b shows that as *Z_start_* increases from 0 to 1.2 mm, the HAZ width at the entrance slightly increases to 11.8 μm. However, when *Z_start_* increases to 1.6 mm, the HAZ width rises sharply to 19.3 μm. With the decrease in *Z_start_*, the piercing time is reduced from 76.2 s to 47.0 s, indicating a continuous improvement in piercing efficiency. Clearly, *Z_start_* = 0.8 mm is the preferred value.

As *Z_start_* moves downward, the piercing efficiency continuously improves, which is primarily attributed to two factors. Firstly, more laser energy is deposited in the middle and lower regions of the hole, promoting the effective expulsion of ablated material from the hole. Secondly, when WD is 30 mm, the laser scanning trajectory exhibits a slight inverted cone shape. The results in Figure 9c and Figure 10a indicate that lowering *Z_start_* leads to more concentrated deposition of laser energy in the central region of the hole.

When *Z_start_* increases from 0 to 0.8 mm, the gradual increase in exit diameter can be attributed to the improvement in piercing efficiency. However, when *Z_start_* exceeds 1.6 mm, the laser spot size at the entrance expands rapidly (exceeding the Rayleigh length), leading to a sharp decrease in laser energy density. Particularly in the region near the hole edge, the larger scanning radius reduces the pulse overlap ratio, thereby weakening the ablation efficiency. The combined effect of these two factors causes rapid contraction of the hole diameter at the entrance, which obstructs and scatters the laser beam, thereby reducing the exit diameter. By measuring the axial cross-section of the hole processed at *Z_start_* = 1.6 mm (as shown in Figure 12), this mechanism is confirmed: within a depth of 580 μm from the entrance, the hole diameter rapidly decreases from 395 μm to 260 μm.

When *Z_start_* varies within the range of 0 to 1.2 mm, as it is less or nearly equal to the Rayleigh length, the focal spot area near the top surface increases slightly. The processing efficiency at the entrance section decreases marginally, while the energy deposition and thermal effects on the top surface increase slightly, leading to a minor increase in the HAZ width. However, when *Z_start_* exceeds 1.6 mm, it surpasses the Rayleigh length of the laser focus. The quick increase in beam diameter leads to significant tapering at the entrance section. Substantial laser energy is deposited near the entrance, and the accumulated thermal effects cause a rapid increase in the HAZ width.

Based on the criteria of minimum HAZ width, maximum exit diameter, and highest piercing efficiency, the selected parameters for the first-stage processing were as follows: the pulse repetition frequency was set to 10 kHz, *Z_start_* was 0.8 mm, the WD of the SSMH was 30 mm. Figure 13 shows the morphology of the hole entrance and exit after the first-stage processing of 100 s. The hole exhibited an entrance diameter of 395.5 ±1.9 μm, an exit diameter of 265.3 ±2.9 μm, a HAZ width of 11.6 ±0.6 μm, and a piercing time of 62.0 ± 0.6 s.

### 3.2. Step 2: High-Quality, Near-Zero Taper Circular Holes Processed Using Half Spiral Scanning

The objective of Step 2 is to process near-zero taper, high-quality circular holes based on the conical holes generated in Step 1. According to the exit diameter and the target aperture, a half-spiral drilling strategy was adopted in Step 2, as illustrated in Figure 3. The outer and inner diameters of the scanning path were 400 μm and 225 μm, respectively. The starting position of the laser focus, *Z_start_*, was maintained at 0.8 mm, and the terminal position at the lower surface of the CFRP plate, i.e., *Z_end_* = 2.0 mm. The feed rate was set to 0.015 mm/s, and the processing time was 80 s.

In this subsection, the effects of pulse repetition frequency and WD on the hole quality were systematically investigated.

#### 3.2.1. Effects of Pulse Repetition Frequency and WD on the Entrance and Exit Morphology

Figure 14, Figure 15 and Figure 16 present the morphological characteristics of micro-holes processed under different pulse repetition frequencies and WD. The results in Figure 14 show that as the repetition frequency increased from 10 kHz to 30 kHz, only slight melting and ablation of the surface resin layer occurred at the hole entrance, resulting in very small HAZ widths, with average values of 7.9 ± 1.8 μm, 8.1 ± 1.7 μm, and 10.6 ± 2.0 μm, respectively. However, as the repetition frequency further increased to 40 kHz, resin ablation became more pronounced, and the HAZ width increased to 14.3 ± 2.3 μm.

The thermal effects at the entrance during the second step were much less than those observed in the first step. As shown in Figure 5 and Figure 6 for the first-step processing, for a pulse repetition frequency of 20 kHz, the HAZ width is as high as 51.4 μm. When the repetition frequency exceeds 30 kHz, the HAZ width even surpasses 150 μm. The reason is that during the first-step processing, the micro-hole has not yet fully penetrated. The ablated material is primarily expelled backward through the entrance via ejection, and the high-pressure airflow disturbance effect inside the blind hole is highly pronounced, leading to severe thermal effects at the entrance region. However, during the second-step processing, since a through-hole has already formed, high-pressure assist gas can flow very quickly through the micro-hole, efficiently carrying away the ablated material via the exit. This change fundamentally alters the flow characteristics of both the assist gas and the ablated material, as well as the heat dissipation pathways, thereby significantly enhancing the cooling efficiency and depressing the thermal effects.

As shown in Figure 14 and Figure 16, when the pulse repetition frequency was increased from 10 kHz to 40 kHz, the entrance diameter of the micro-hole increased by only 5 μm. In contrast, the exit diameter expanded significantly from 344 μm to 424 μm. Notably, when the repetition frequency was raised from 10 kHz to 20 kHz, the exit diameter grew rapidly to 379.5 ± 2.3 μm, corresponding to an increment of 36 μm. Furthermore, Figure 14 clearly demonstrates that at a repetition frequency of 10 kHz, the thermal effect at the outlet was minimal. The resin around the edge remained well, with no evident melting. There was only occasional small spalling, with a size less than 5 μm. However, due to the strong scattering of the laser beam by the hole wall, the edge processing was insufficient, resulting in a low circularity of only 92.9%. Irregular concave-convex structures were observed along the edge, as illustrated in (d1). When the pulse repetition frequency was increased to 20 kHz, the exit edge became smoother and more regular, with the circularity improving to 96.3%. Very slight ablation and spalling (less than 5 μm in size) of the resin were observed near the edge. Unlike the situation at the entrance, no intense resin ablation leading to exposure of the underlying carbon fibers was observed. As the repetition frequency was further increased to 40 kHz, the exit shape remained regular. However, the area of resin ablation and spalling at the edge expanded noticeably, exceeding 6 μm in size.

The results indicate that appropriately increasing the pulse repetition frequency plays a crucial role in enhancing the exit diameter and quality of deep micro-holes. This is primarily attributed to the lower processing efficiency at the exit edge region, which stems from two main factors: first, significant scattering of the laser beam by the hole wall leads to a reduction in laser intensity; second, the lower pulse overlap ratio around the hole periphery decreases material removal efficiency.

Figure 15 presents the morphology of micro-holes processed by a 20 kHz laser. As the WD increases from 40 mm to 55 mm, the exit diameter expands from 296.6 ± 5.5 μm to 379.5 ± 2.3 μm, whereas the entrance diameter slightly decreases from 405 μm to 400 μm. The HAZ width shows minimal variation, fluctuating within the range of 6–9 μm.

Figure 16d shows the taper of deep micro-holes processed at different repetition frequencies and WD. For lasers with repetition frequencies of 30 kHz and 40 kHz, the tapers are all less than ±0.007 when the WD is 50 mm and 55 mm, respectively. These holes can be considered near-zero taper circular holes. For the laser with a repetition frequency of 20 kHz, the taper is extremely small, measuring only 0.0045 ± 0.0005 at a WD of 55 mm. However, for the laser with a repetition frequency of 10 kHz, the taper remains as high as 0.0130 ± 0.0011, even when the WD is adjusted to its maximum value of 55 mm.

Based on a comprehensive analysis of the entrance HAZ width, exit morphology, and taper, the selected parameters were determined to be a pulse repetition frequency of 20 or 30 kHz with a WD of 55 mm.

#### 3.2.2. Effects of Pulse Repetition Frequency and WD on the Surface Quality of the Inner Wall

To investigate the effects of pulse repetition frequency and WD on the surface quality of the inner wall, the micro-holes were first sectioned axially and polished. Subsequently, the inner wall surfaces were examined and analyzed using methods such as SEM, COM, and Raman spectroscopy.

Under the condition where the pulse repetition frequency was fixed at 20 kHz and the WD was varied from 40 mm to 55 mm, observation revealed that, apart from a significant increase in the exit diameter, there were no apparent changes in the morphological regularity, surface roughness, or chemical composition of the inner wall. This indicates that the WD has a minor influence on the surface quality.

In the following, this study will focus on investigating the effect of pulse repetition frequency on the surface quality of the inner wall.

Figure 17 presents the inner wall morphology of micro-holes processed as the pulse repetition frequency increased from 10 kHz to 30 kHz, with the WD maintained at 55 mm. The overall profiles indicate that the inner walls are very regular, with no evidence of resin melting, ablation, or other damage phenomena on the outer sides of the wall. The field-of-view positions of images Figure 17(a2–c2) differ from one another, which is attributed to the non-uniform weaving of the CFRP plate; nevertheless, all images were deliberately selected in the vicinity of the interlayer interfaces. These images clearly demonstrate that the parallel-aligned and perpendicular-aligned fiber layers remain well bonded, with no significant delamination being observed.

Figure 17(a3–c3) present locally magnified images of the fiber layers aligned perpendicular to the bottom of the inner wall. When the pulse repetition frequency was in the range of 10–20 kHz, the vertically aligned fibers and the resin matrix were tightly bonded, resulting in a very flat surface, except for occasional micro-pits formed due to resin ablation [21]. As the pulse repetition frequency increased to 30 kHz, the resin at the ends of some fibers was ablated, leaving separated fiber ends [39,50]. The experimental results indicate that higher pulse repetition frequencies lead to more intense resin ablation, resulting in a greater number of longer, exposed, and separated fiber ends.

When the pulse repetition frequency ranged from 10 to 30 kHz, the resin and fibers within the parallel-aligned fiber layers remained strongly bonded, with almost no gaps observed between the fibers, and no evidence of fiber ablation or detachment. This can primarily be attributed to the fact that the thermal conductivity along the radial direction of the fibers is only about 1/10 of that along the axial direction, thereby significantly reducing the thermal effects.

The three-dimensional topography of the inner wall was measured using COM, and the surface roughness was analyzed. Figure 18(a1–c1) show the overall COM morphology of the inner wall for pulse repetition frequencies of 10, 20, and 30 kHz. To more clearly analyze the effect of pulse repetition frequencies on the surface quality of fiber layers with varying orientations, a 150 μm × 100 μm area was selected on the fiber layers oriented perpendicular to and parallel to the inner wall bottom, respectively, for high-magnification COM observation. Both the top and bottom surface layers of the specimen consist of woven carbon fiber fabric, with a single-layer thickness of approximately 230 μm. For both the top and bottom surface layers, two regions of 150 μm × 100 μm area were selected for measurement, respectively. One 150 μm × 100 μm area was selected from each of the internal 11 plies for measurement.

Figure 18(a2–c2) indicate that at pulse repetition frequencies of 10 and 20 kHz, the surface of the fiber layer perpendicular to the inner wall is very smooth, with no significant pits. However, when the repetition frequency increases to 30 kHz, both the number and depth of micro-pits increase significantly. Three lines spaced at 20 μm intervals were selected in the bottom region for roughness measurement, with the results shown in Figure 18(a3–c3). At pulse repetition frequencies of 10 and 20 kHz, the surface is extremely smooth, with roughness values of only 0.245 ± 0.114 μm and 0.257 ± 0.030 μm, respectively. However, when the repetition frequency increases to 30 kHz and 40 kHz, the roughness rises rapidly to 0.455 ± 0.161 μm and 1.239 ± 0.118 μm, respectively. In this study, the roughness parameter Pa was calculated directly from the primary profile without filtering. The profile includes surface waviness and even form deviations, which leads to an overestimated roughness value. The roughness of all seven perpendicular layers was measured. The results show that, at laser repetition rates of 10–40 kHz, the average values Pa for each hole are 0.226 ± 0.087 μm, 0.244 ± 0.116 μm, 0.446 ± 0.055 μm, and 0.961 ± 0.231 μm, respectively. The trend of average roughness variation with laser frequency is shown in Figure 19.

For the fiber layer parallel to the inner wall bottom, Figure 18(a4–c4) reveal that the inner wall surface is very regular. Occasionally, severed fiber segments approximately 30–60 µm in length detach from the hole wall, leaving behind cracks with widths of about 3–6 µm and depths of about 1.5–3.0 µm. Unlike the perpendicular case, the roughness of the parallel fiber layer fluctuates between 0.49 µm and 0.57 µm, showing no clear dependence on the pulse repetition frequency; instead, it exhibits a more pronounced dependence on the fracture, detachment, and tight arrangement of the parallel fibers.

In summary, the measurement and analysis results from both SEM and COM indicate that the inner walls processed with 10–20 kHz lasers are regular and smooth.

Surface roughness is an effective parameter for characterizing short-range surface undulations. However, this parameter cannot accurately reflect the height difference between the fiber ends and the surrounding resin, namely the exposed length of the fibers. This parameter is crucial for evaluating the inner wall quality of micro-holes, as it directly reflects the extent of damage to the strength of CFRP caused by laser processing. Taking the inner wall of a micro-hole processed with a 20 kHz laser as an example, typical areas were selected from fiber layers perpendicular and parallel to the inner wall bottom, respectively, to measure and analyze the height difference between the fibers and the adjacent resin. As shown in Figure 20a, three representative groups of fibers were selected for analysis: Group 1 (a–e) consists of densely arranged fibers, Group 2 (h–l) comprises typically arranged fibers, and Group 3 (1–5) includes fibers adjacent to micro-pits. For each fiber end (as illustrated in the enlarged view labeled “j”), the fiber height was measured within the red circular area, while the average height of the surrounding resin was measured along the white circular path. The difference between these two measurements corresponds to the exposed fiber length. Figure 20c presents the statistical results of the height differences for the three groups: the maximum exposed length is only 0.51 μm, the minimum value is −0.26 μm, and the average value is very small, merely 0.155 μm.

Figure 20b,d present and analyze the height differences between the cross-sections of parallel-aligned fibers and the adjacent resin. For each fiber cross-section, following the method shown in the enlarged view labeled “j”, the height of the fiber profile was measured within the red box, while the heights along the two white line segments were measured to determine the average resin height in the two gaps. The average of these two measured values was taken as the mean height of the inter-fiber resin. The height difference between the fiber profile and the resin represents the exposed height of the fiber cross-section. The statistical mean value across the three groups is extremely small, measuring only 0.48 μm.

These results clearly demonstrate that, despite the vaporization temperature of carbon fibers being 3200 K higher than the thermal decomposition temperature of resin, thermal effects are effectively suppressed during the two-step laser processing at 20 kHz. The fibers in the inner wall remain well-bonded to the resin, with an average exposed height of the fiber ends/cross-sections of only 0.32 μm.

Damage to the lattice structure can affect the strength and toughness of carbon fibers, thereby influencing the performance of CFRP components. To further investigate the lattice damage of carbon fibers after laser processing at different repetition frequencies, Raman spectroscopy was performed on both the original (unprocessed) surface and the vertical fiber layers in the inner wall. A 532 nm laser was focused onto the center of the fiber end using a 50× objective lens to excite the Raman spectra.

Figure 21 shows that all Raman spectra contain the D band (~1355 cm^−1^) and the G band (~1593 cm^−1^). The *I_D_*/*I_G_* ratio of the carbon fibers without laser processing was 0.919. As the pulse repetition frequency increased, the intensity of the D band gradually enhanced. At pulse repetition frequencies of 10 kHz and 20 kHz, the *I_D_*/*I_G_* ratio slightly increased to 0.933 and 0.934, respectively. However, when the repetition frequency was further raised to 30 kHz and 40 kHz, the *I_D_*/*I_G_* ratio rapidly increased to 0.967 and 0.986. The D band is associated with lattice defects in carbon fibers [51,52]. The enhancement of the D band and the rise in the *I_D_*/*I_G_* ratio indicate that during laser processing, higher repetition frequencies led to the formation of more disordered structures at the fiber ends, accompanied by an increase in defect density. These results demonstrate that in the micro-holes processed at a repetition frequency of 20 kHz, the *I_D_*/*I_G_* ratio of the carbon fibers on the inner wall increased by only 1.6%, indicating that the lattice structure remained well preserved, without significant lattice defects.

Based on a comprehensive analysis of the entrance and exit morphology, taper, as well as the regularity, roughness, and lattice defects of the inner wall surface of the deep micro-holes, the processing quality achieved at 20 kHz is superior to that at other investigated frequencies.

To further investigate the consistency and stability of the two-step drilling method and the picosecond fiber laser processing system, a 10 × 10 array of deep micro-holes with a spacing of 1 mm was fabricated on a 15 × 15 × 2 mm^3^ plate (as shown in Figure 22). The entrance and exit morphologies of the micro-holes were measured using a high-magnification optical microscope. The results demonstrated very good consistency in hole diameter, measuring 400 ± 25 μm, high roundness exceeding 96% in all cases, and minimal thermal impact with the HAZ width consistently below 10 μm. These findings indicate that the method and system exhibit promising potential for industrial applications.

## 4. Conclusions

In this study, a high-pressure gas-assisted picosecond fiber laser spiral drilling system was established. By employing a two-step drilling method, high-quality and highly efficient machining of deep micro-holes on CFRP plate was achieved. The main results are summarized as follows.

(1) In the first step, the processing parameters were systematically investigated and determined as follows: a pulse repetition frequency of 10 kHz, a wedge displacement of WD = 30 mm, and a starting position of the laser focus *Z_start_* = 0.8 mm.

Using this set of parameters, a typical tapered hole was fabricated in a 2 mm-thick plate with the following characteristics: an entrance diameter of 395.5 ± 1.9 μm, a HAZ width of 11.6 ± 0.6 μm, an exit diameter of 265.3 ± 2.9 μm, and a processing time of 100 s.

(2) In the second step, the process parameters were determined as follows: a pulse repetition frequency of 20 kHz, a wedge displacement of WD = 55 mm, and a feed rate of 0.015 mm/s.

Using this set of parameters, a typical hole exhibited the following morphology characteristics: an entrance diameter of 400 ± 25 μm, a taper of 0.0045 ± 0.0005, a HAZ width of 8.1 ± 1.7 μm, and a processing time of 80 s.

The inner-wall surface exhibited exceptionally high quality, characterized by an average roughness of 0.379 ± 0.169 μm, indicating a very smooth inner surface; an average exposed height of the fiber ends/cross-sections of 0.32 μm, indicating excellent fiber–resin bonding; and a slight increase of 1.6% in the (*I_D_*/*I_G_*) ratio, suggesting that the lattice structure of the fiber ends was well preserved without significant crystalline defects.

Compared with the results reported in the recent literature (as shown in Table 4), the holes machined using the two-step spiral drilling strategy exhibit superior quality and higher processing efficiency. The picosecond fiber laser processing system developed in this study demonstrates excellent stability and produces holes with high consistency, indicating promising potential for industrial applications.

## Figures and Tables

**Figure 1:**
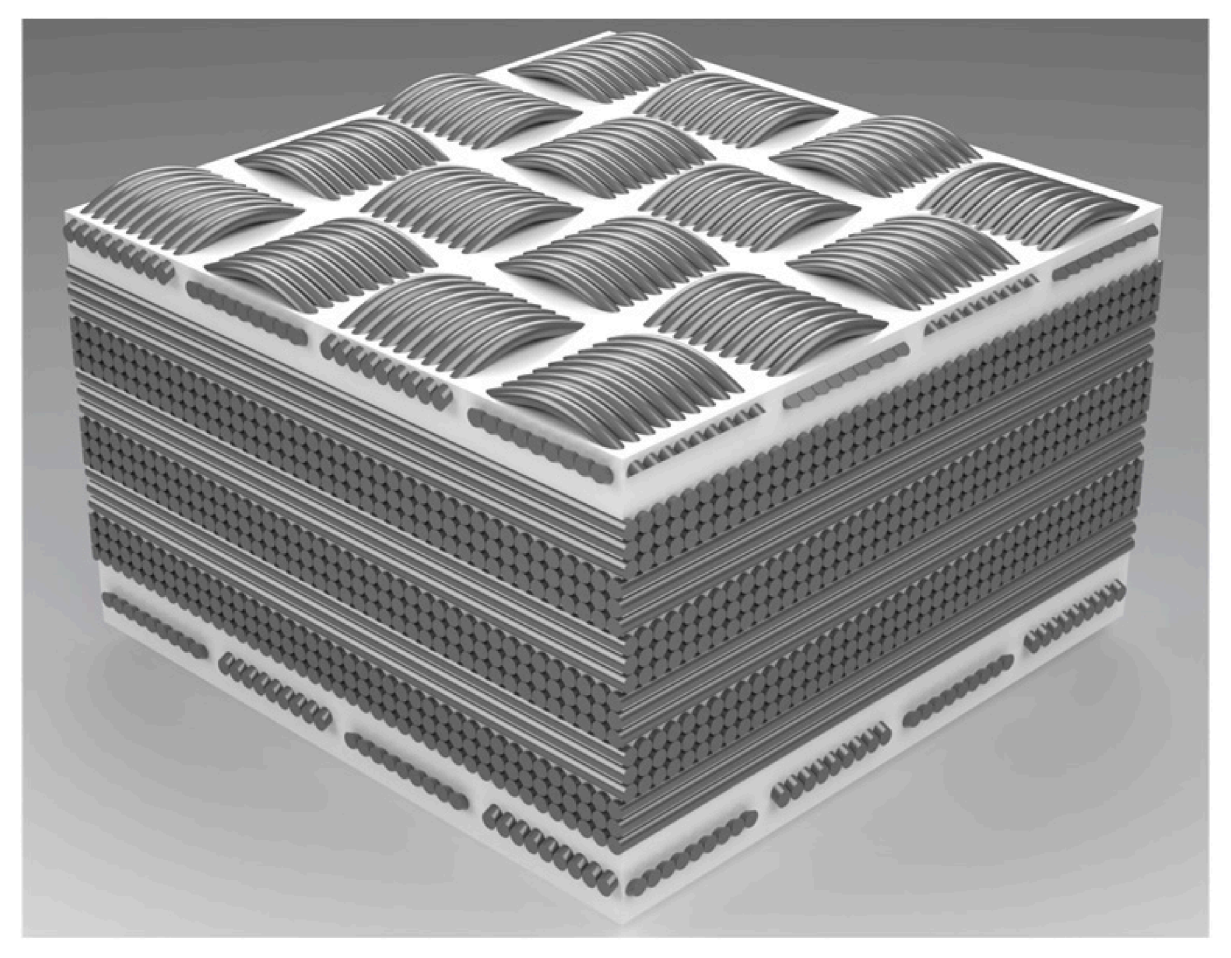
Schematic diagram of a multidirectional CFRP plate.

**Figure 2:**
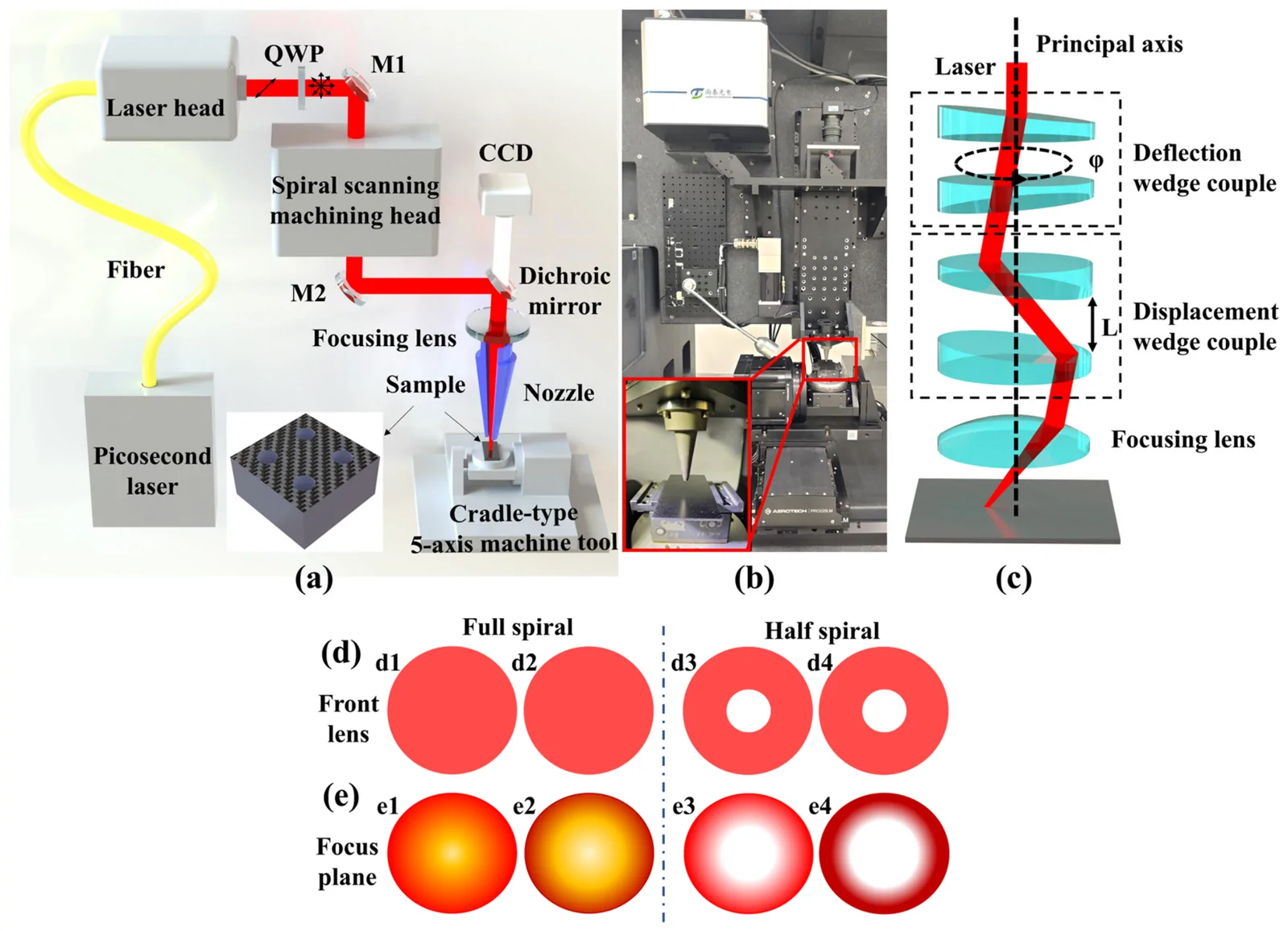
(**a**) Picosecond fiber laser spiral drilling system mounted on a cradle-type five-axis machine tool; (**b**) Photograph of the experimental setup; (**c**) Schematic diagram of the optical path of the SSMH; (**d**,**e**) Schematic diagram of the optical path trajectory before the lens (**d1**–**d4**) and at the focal plane (**e1**–**e4**). Trajectory patterns (**1**) and (**2**) correspond to full spiral scanning, while patterns (**3**) and (**4**) correspond to half spiral scanning. Patterns (**1**) and (**3**) represent conditions with a small WD, whereas patterns (**2**) and (**4**) correspond to a large WD.

**Figure 3:**
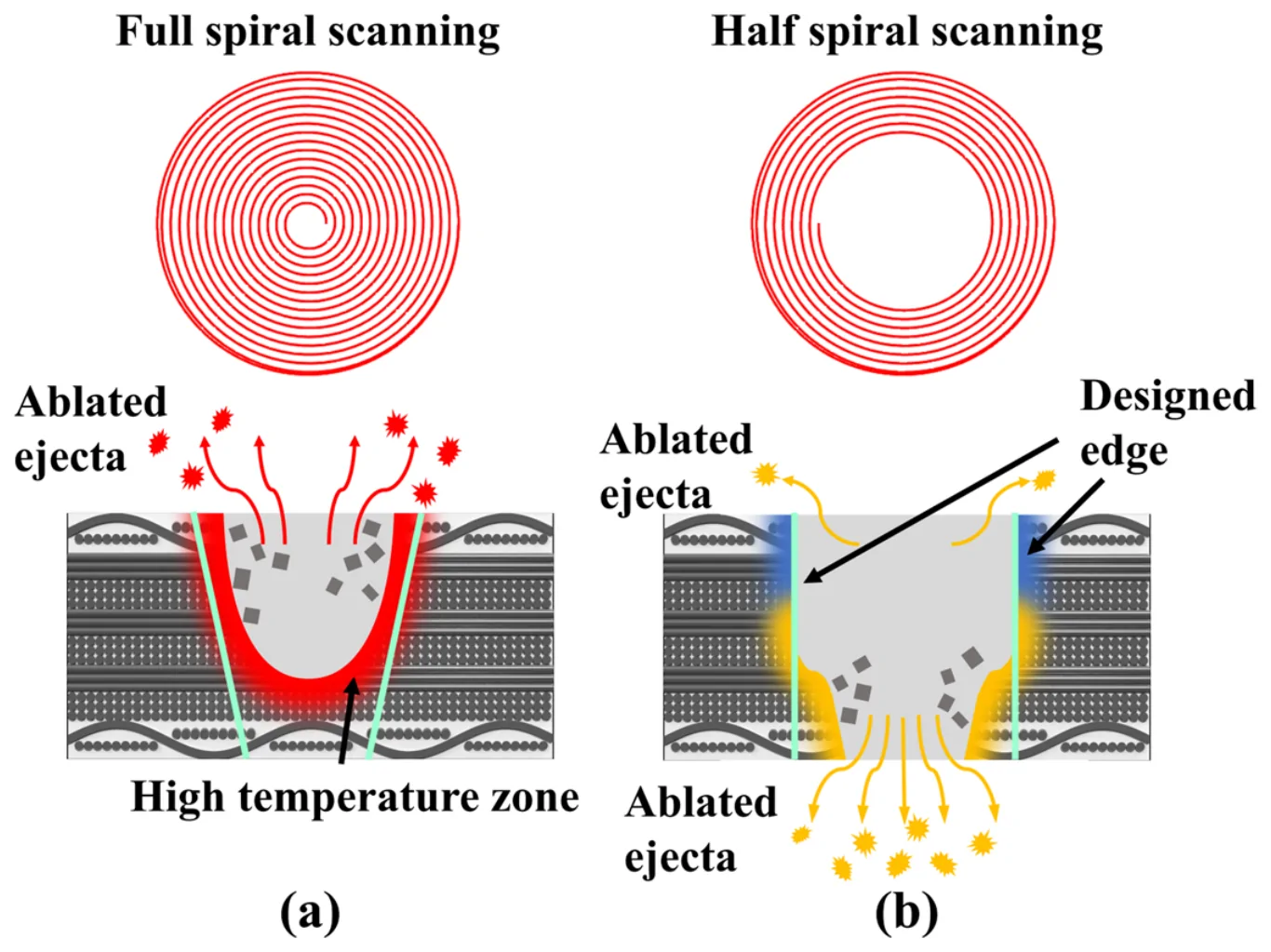
The strategy and method of Step 1 (**a**), and Step 2 (**b**). The red, yellow, and blue colors at the hole edge qualitatively represent high, medium, and low temperatures, respectively.

**Figure 4:**
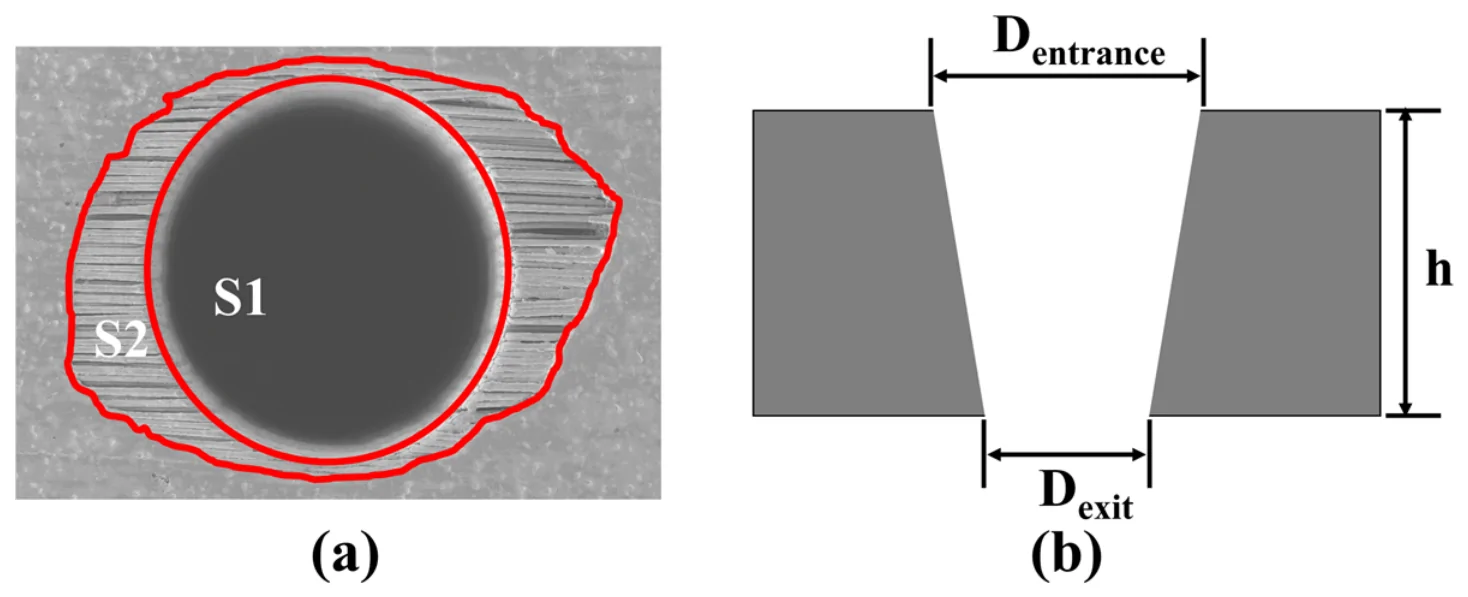
(**a**) Schematic diagram for the measurement and (**b**) characterization of HAZ width and taper.

**Figure 5:**
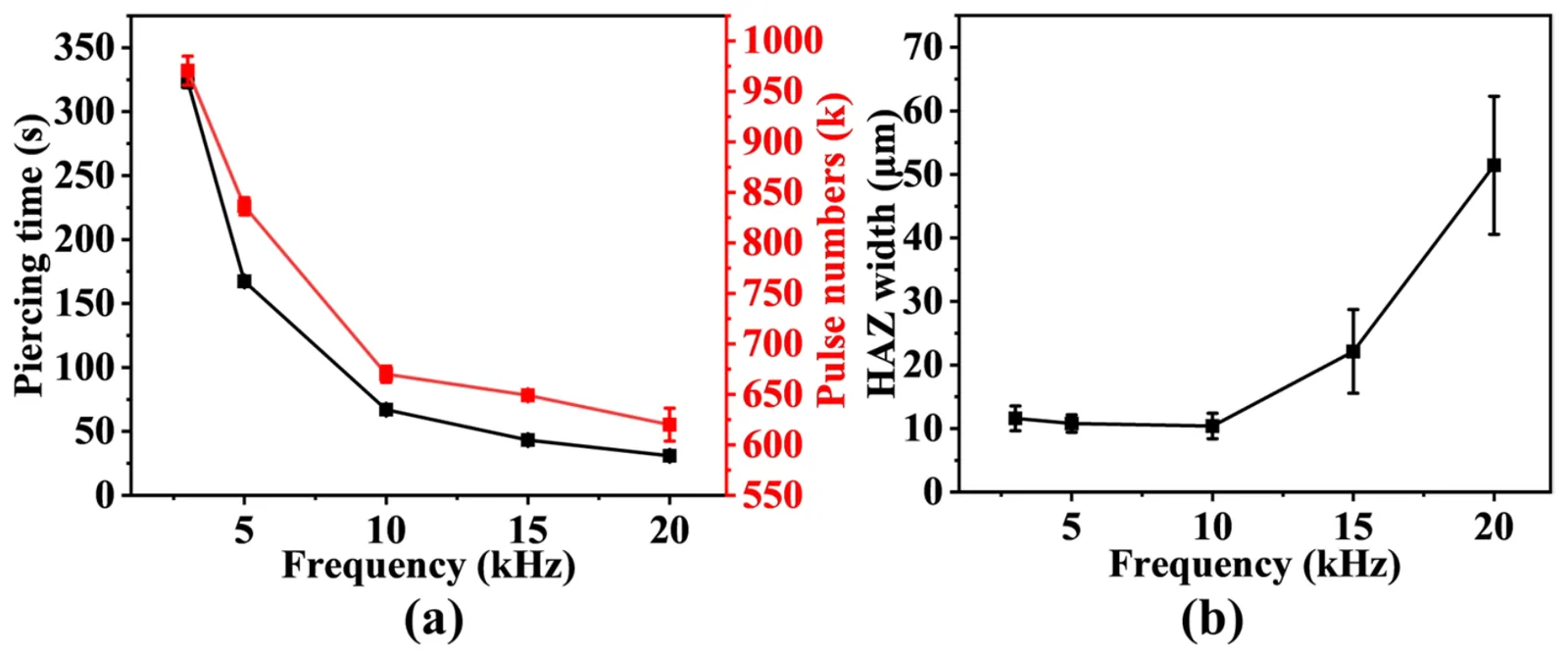
(**a**) Piercing time and cumulative pulse number, and (**b**) HAZ width as a function of pulse repetition frequency.

**Figure 6:**
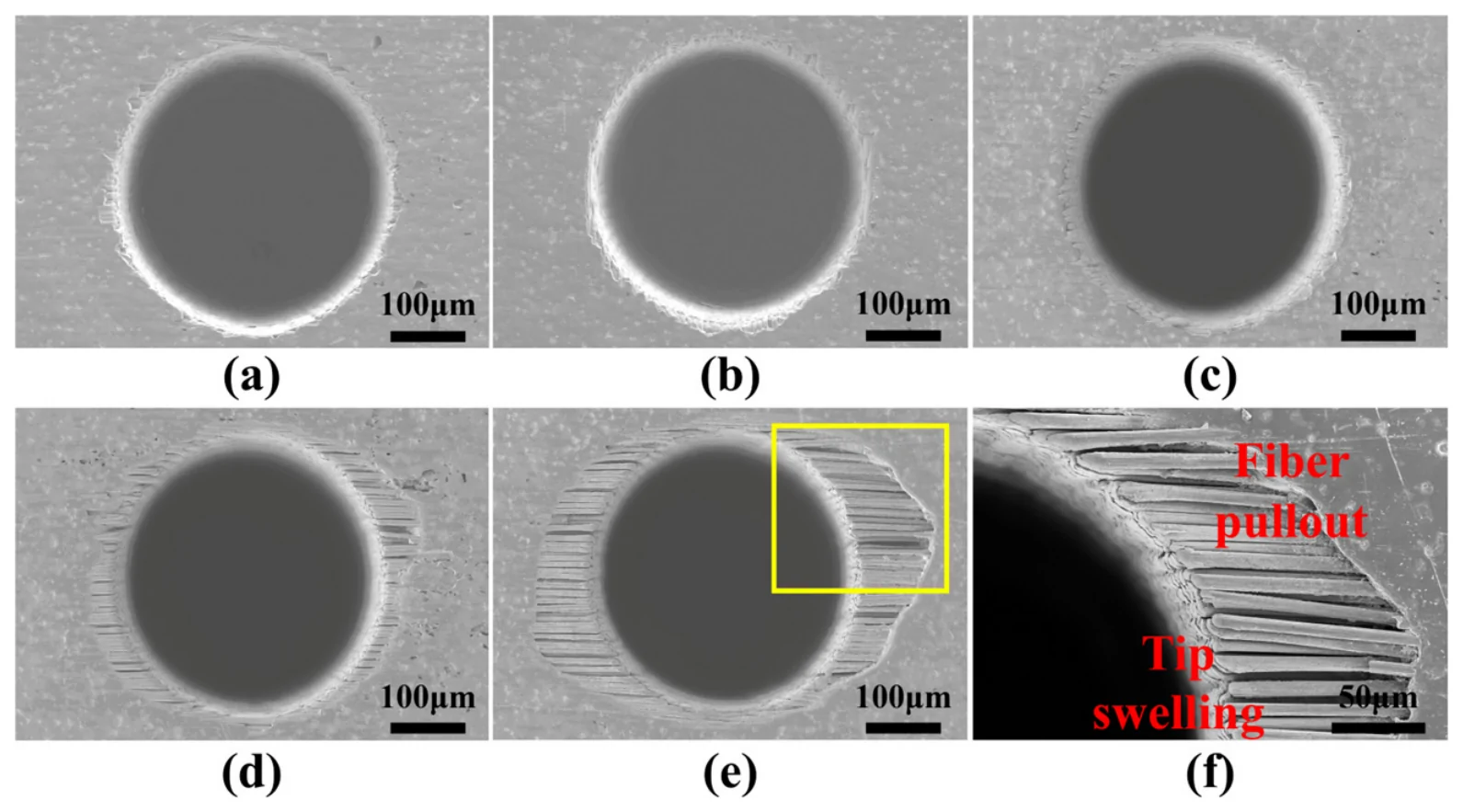
The morphology at the entrance after laser perforation at different repetition frequencies. (**a**–**e**) Pulse repetition frequencies are 3 kHz, 5 kHz, 10 kHz, 15 kHz, and 20 kHz, respectively. (**f**) Magnified view of the region within the yellow square in (**e**).

**Figure 7:**
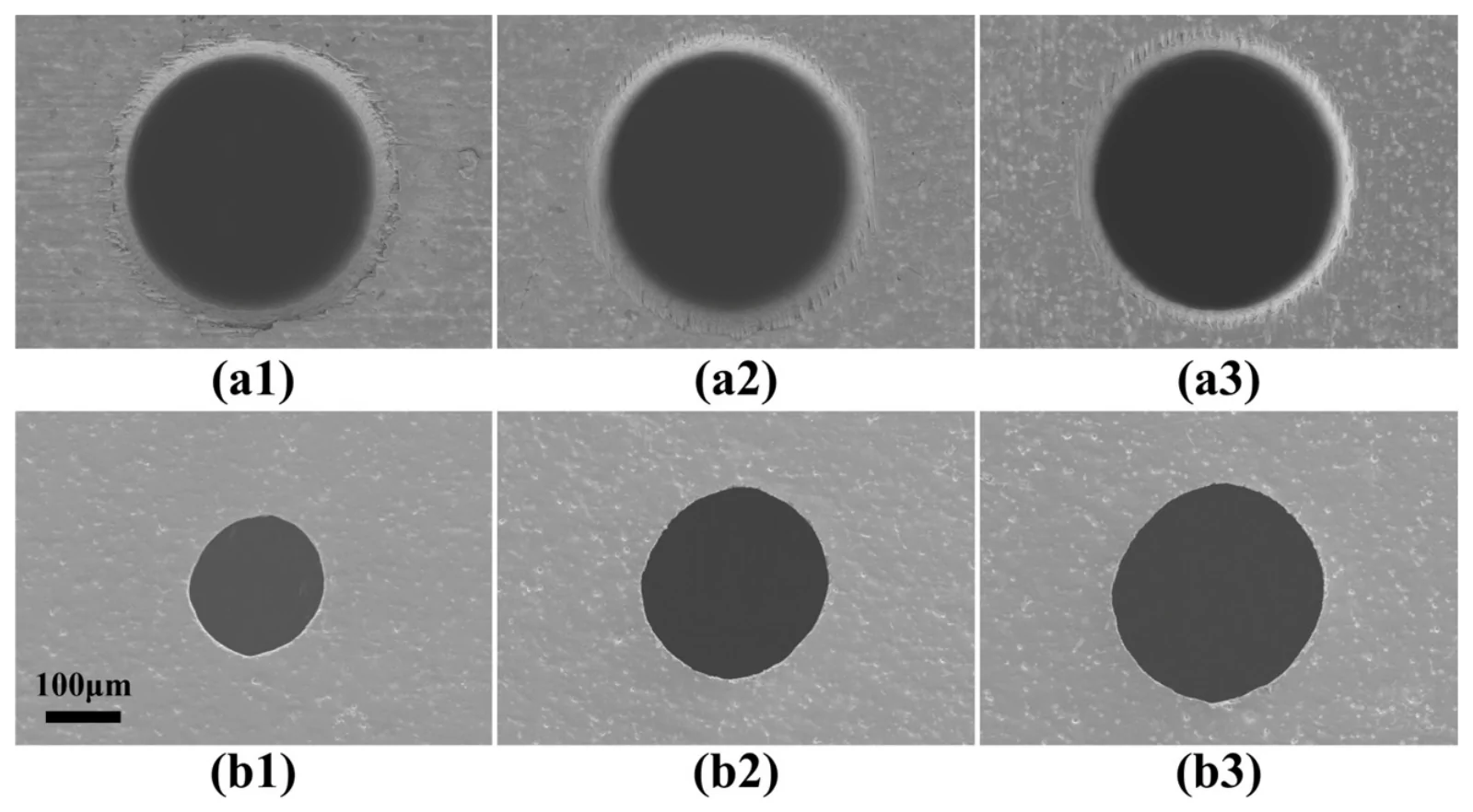
SEM images of the hole entrance (**a1**–**a3**) and exit (**b1**–**b3**) morphologies for WD = 10, 30, and 50 mm (labeled as 1, 2, and 3, respectively).

**Figure 8:**
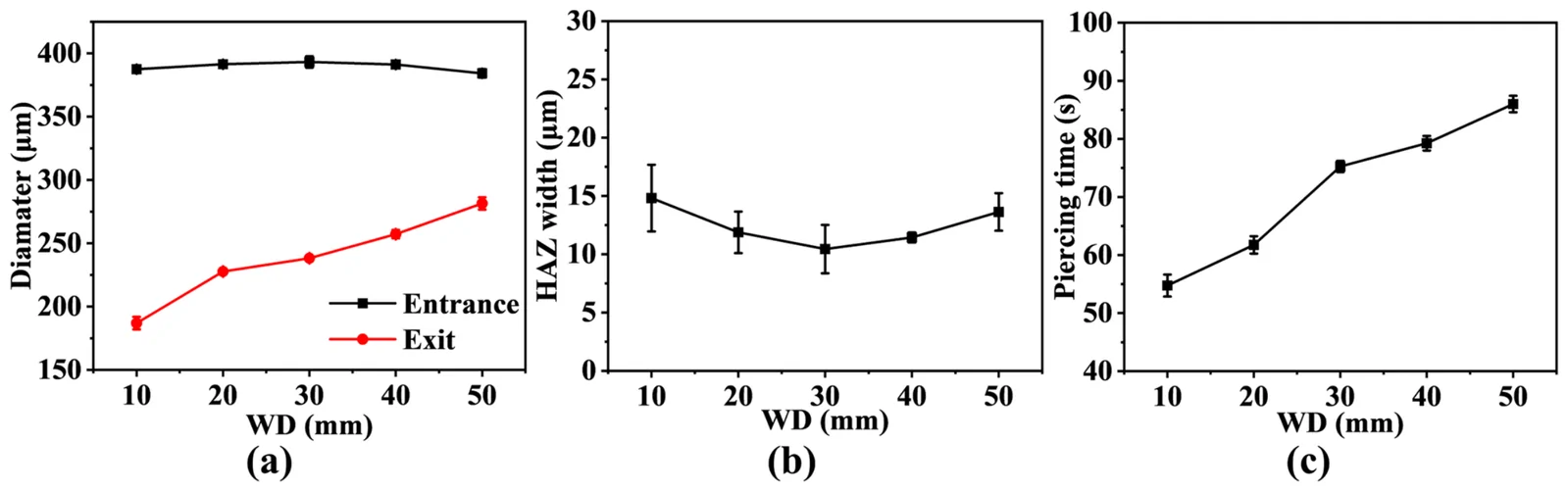
(**a**) Entrance and exit diameters, (**b**) HAZ width, and (**c**) perforation time as functions of WD.

**Figure 9:**
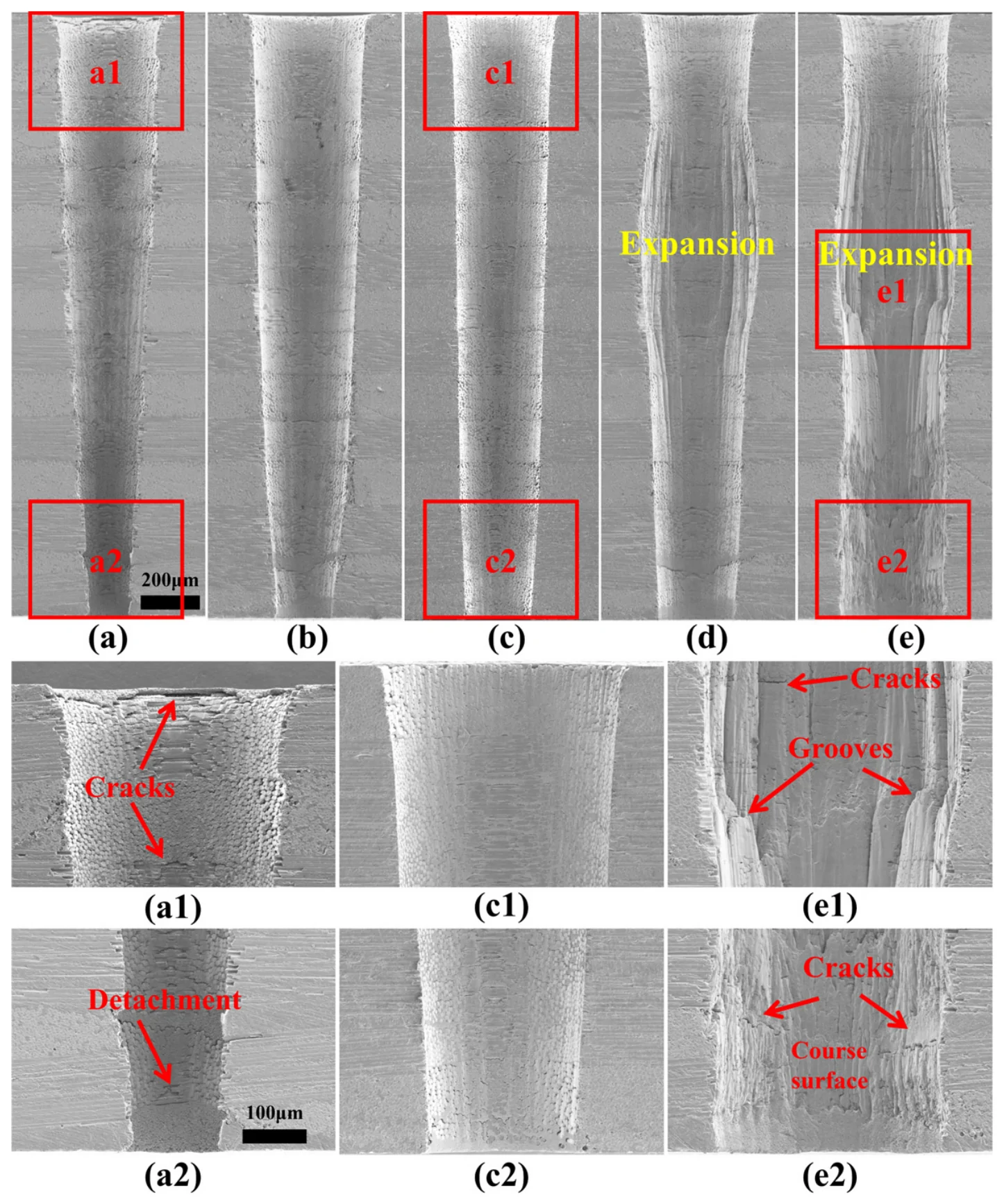
The cross-sectional morphology of the holes processed for 100 s with a feed rate of 0.02 mm/s. (**a**–**e**) WD is 10, 20, 30, 40, and 50 mm, respectively. (**a1**,**c1**,**e1**) and (**a2**,**c2**,**e2**) show the enlarged views of the areas marked by the corresponding squares.

**Figure 10:**
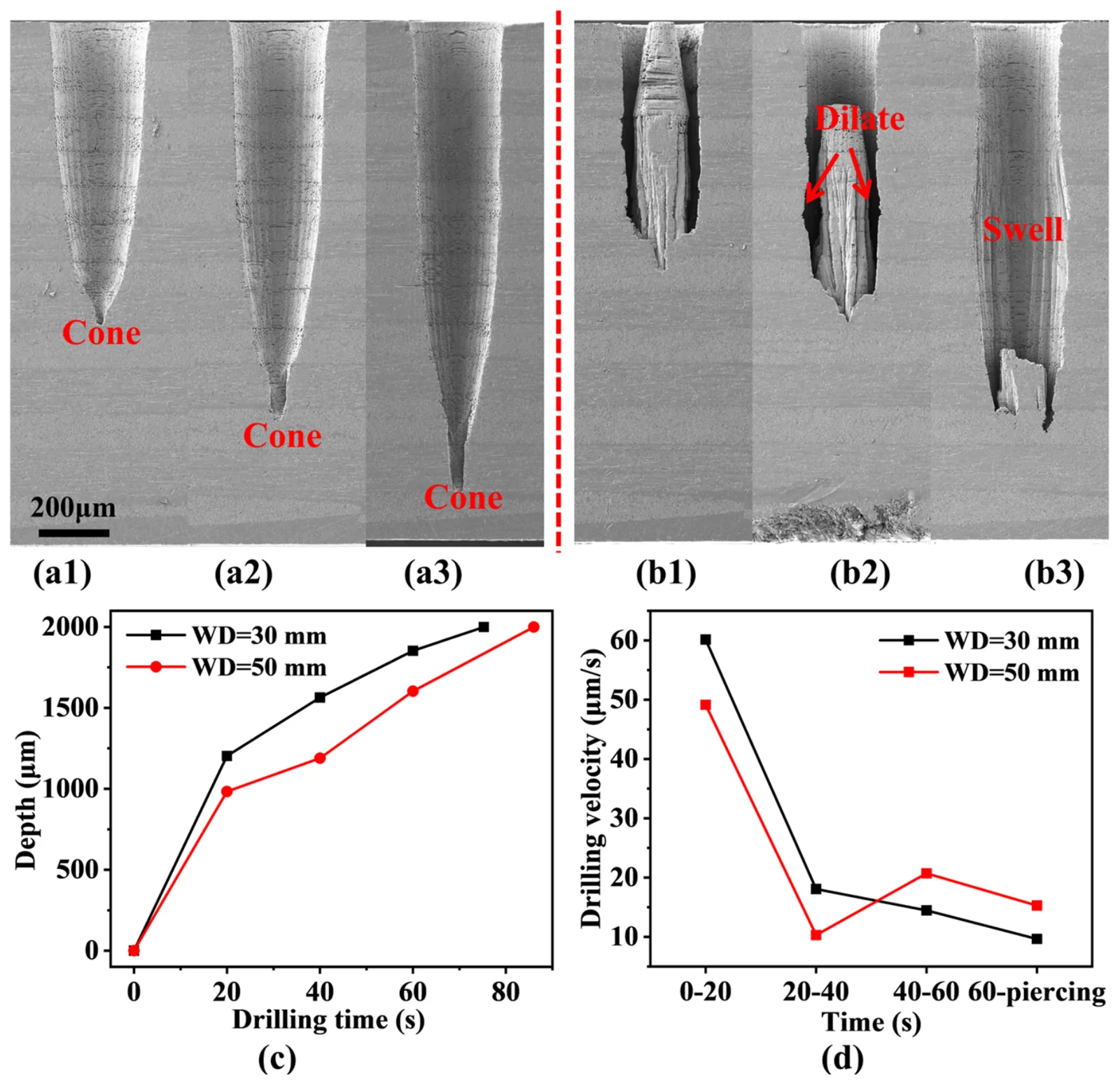
The cross-sectional images of blind holes for different drilling times, with panels (**a**) and (**b**) corresponding to WD = 30 mm and 50 mm, respectively. (**1**–**3**) Processing times are 20 s, 40 s, and 60 s. The feed rate was maintained at 0.02 mm/s for all cases. (**c**) The depth of the blind holes drilled over different time intervals. (**d**) The average drilling speed within each corresponding time interval.

**Figure 11:**
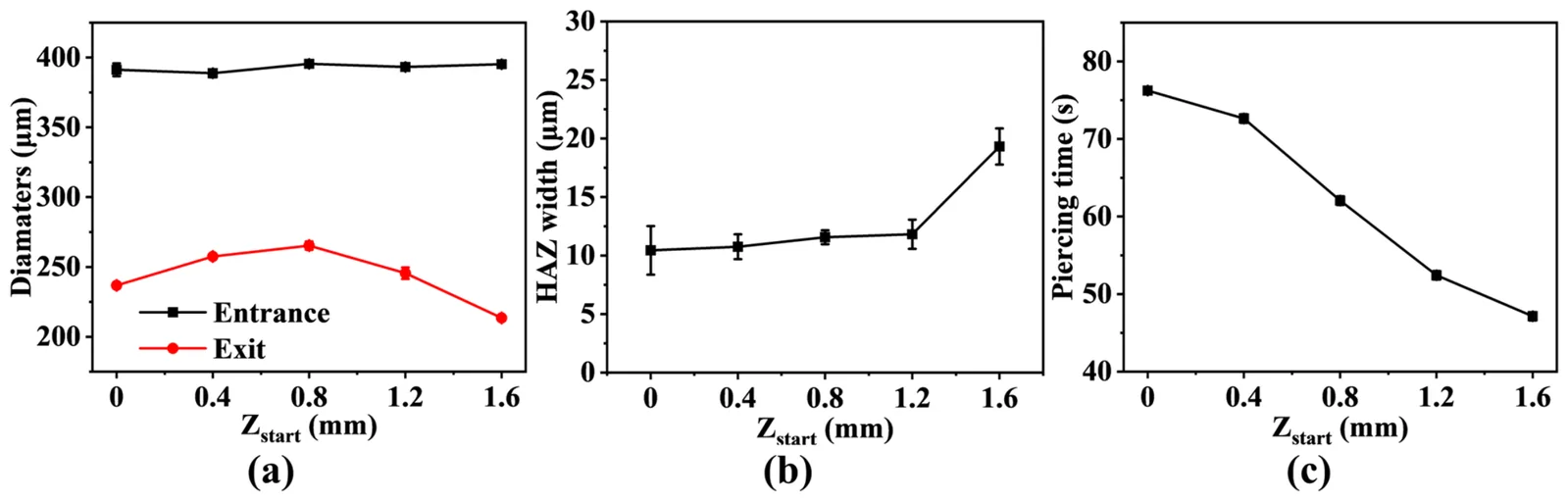
(**a**) Diameter, (**b**) HAZ width, and (**c**) piercing time for holes drilled at different *Z_start_*.

**Figure 12:**
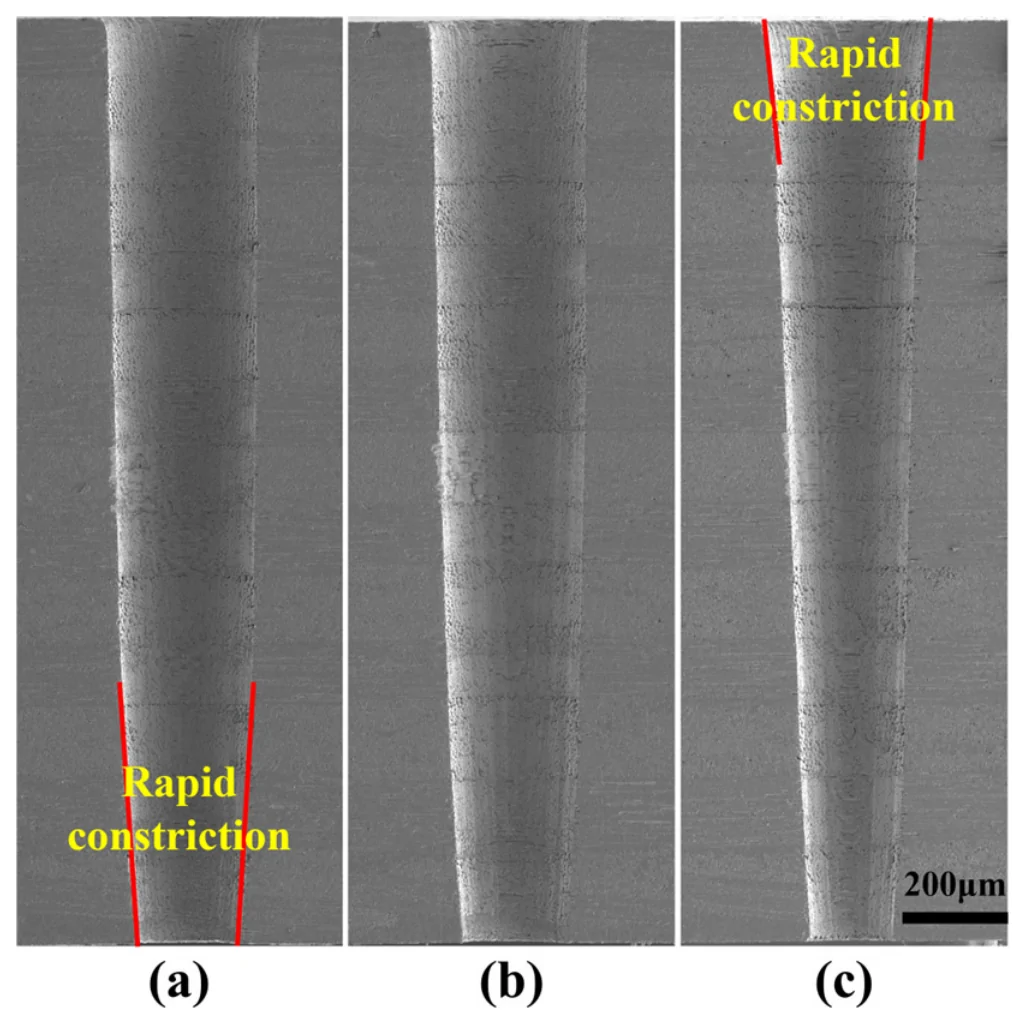
Axial cross-sectional SEM images of holes processed with *Z_start_* of 0.4 mm (**a**), 0.8 mm (**b**), and 1.6 mm (**c**).

**Figure 13:**
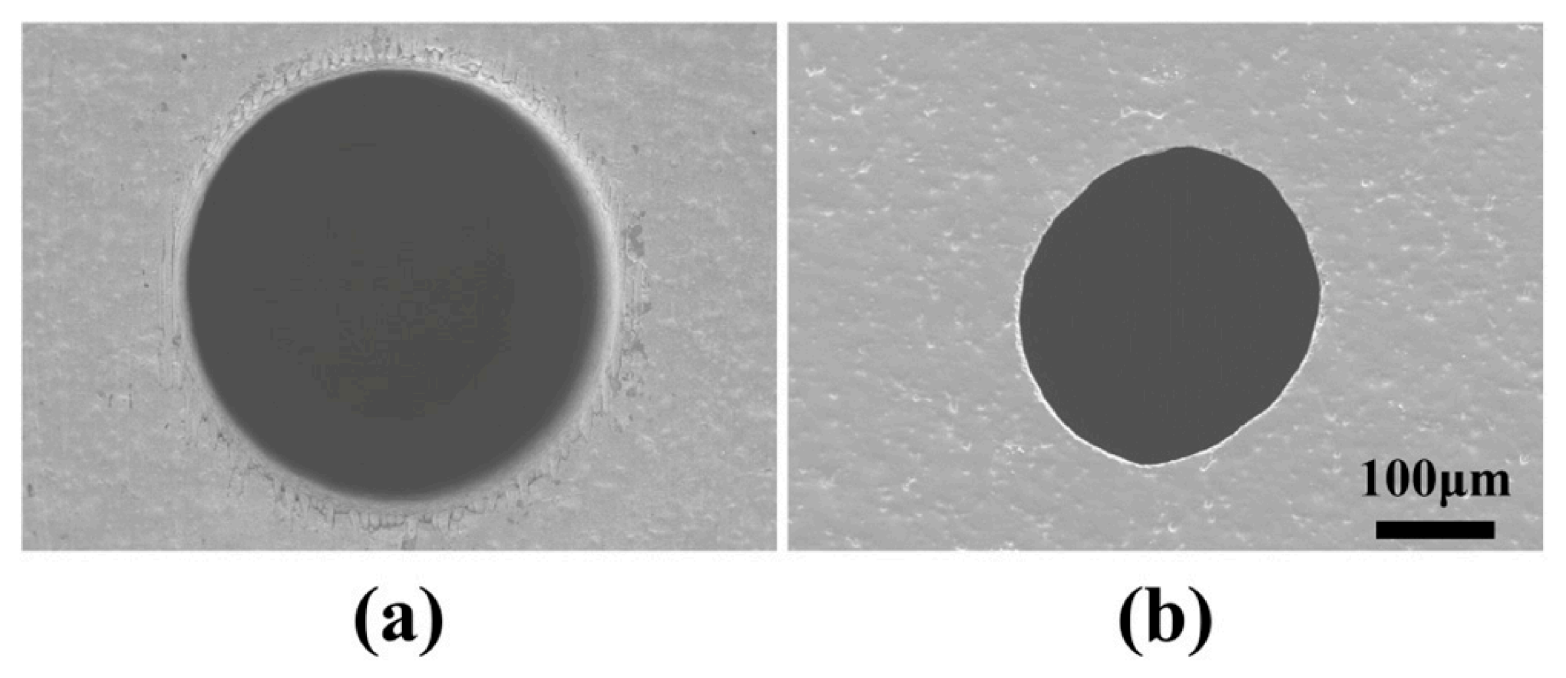
SEM images of the entrance (**a**) and exit (**b**) of the hole after Step 1 processing.

**Figure 14:**
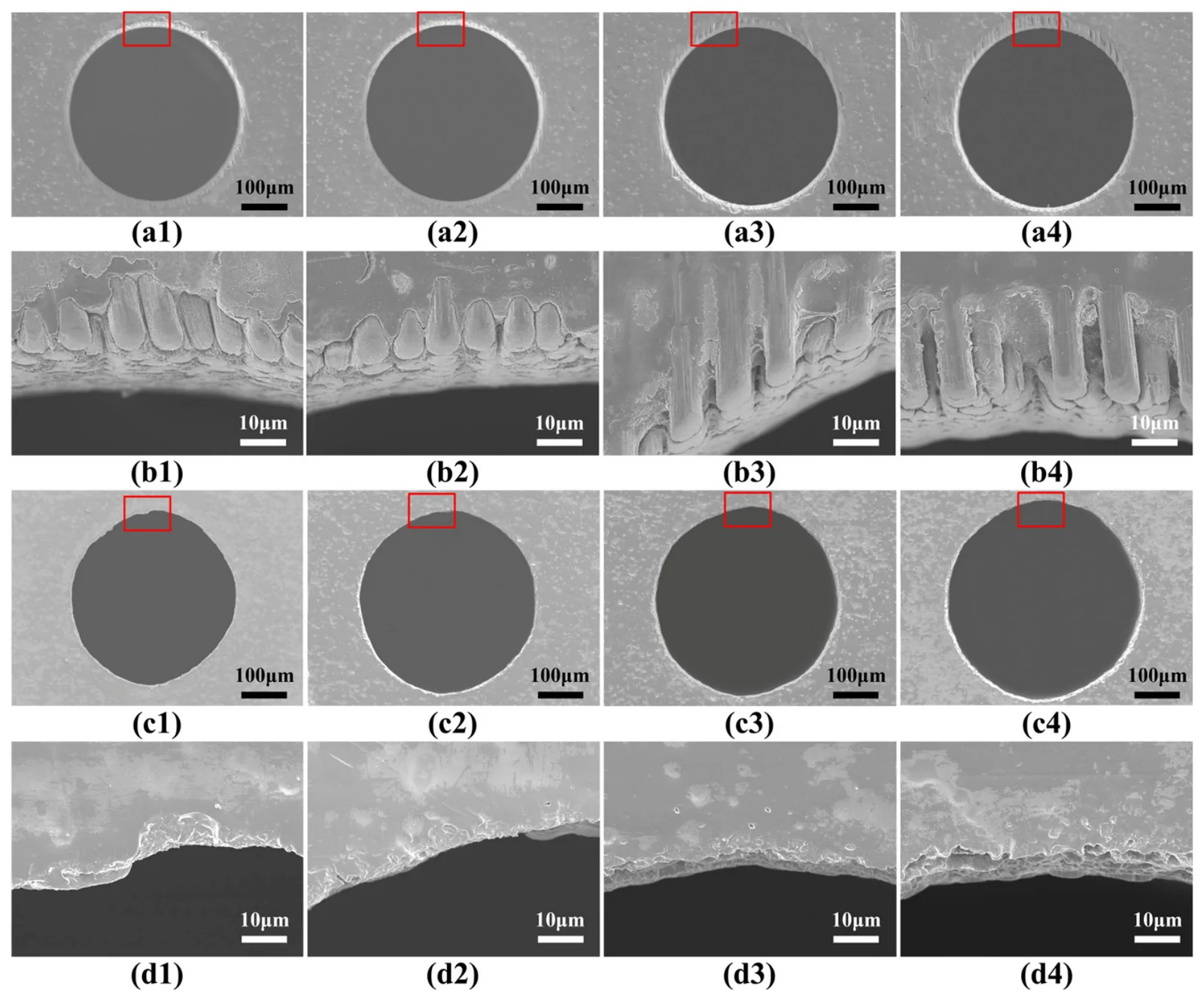
Morphology of the entrance (**a1**–**a4**), (**b1**–**b4**) and exit (**c1**–**c4**), (**d1**–**d4**) after two-step processing with pulse repetition frequencies of 10 kHz, 20 kHz, 30 kHz, and 40 kHz, respectively. (**b1**–**b4**) and (**d1**–**d4**) are enlarged views of the edge regions indicated by the squares in (**a1**–**a4**) and (**c1**–**c4**), respectively. Here, WD = 55 mm.

**Figure 15:**
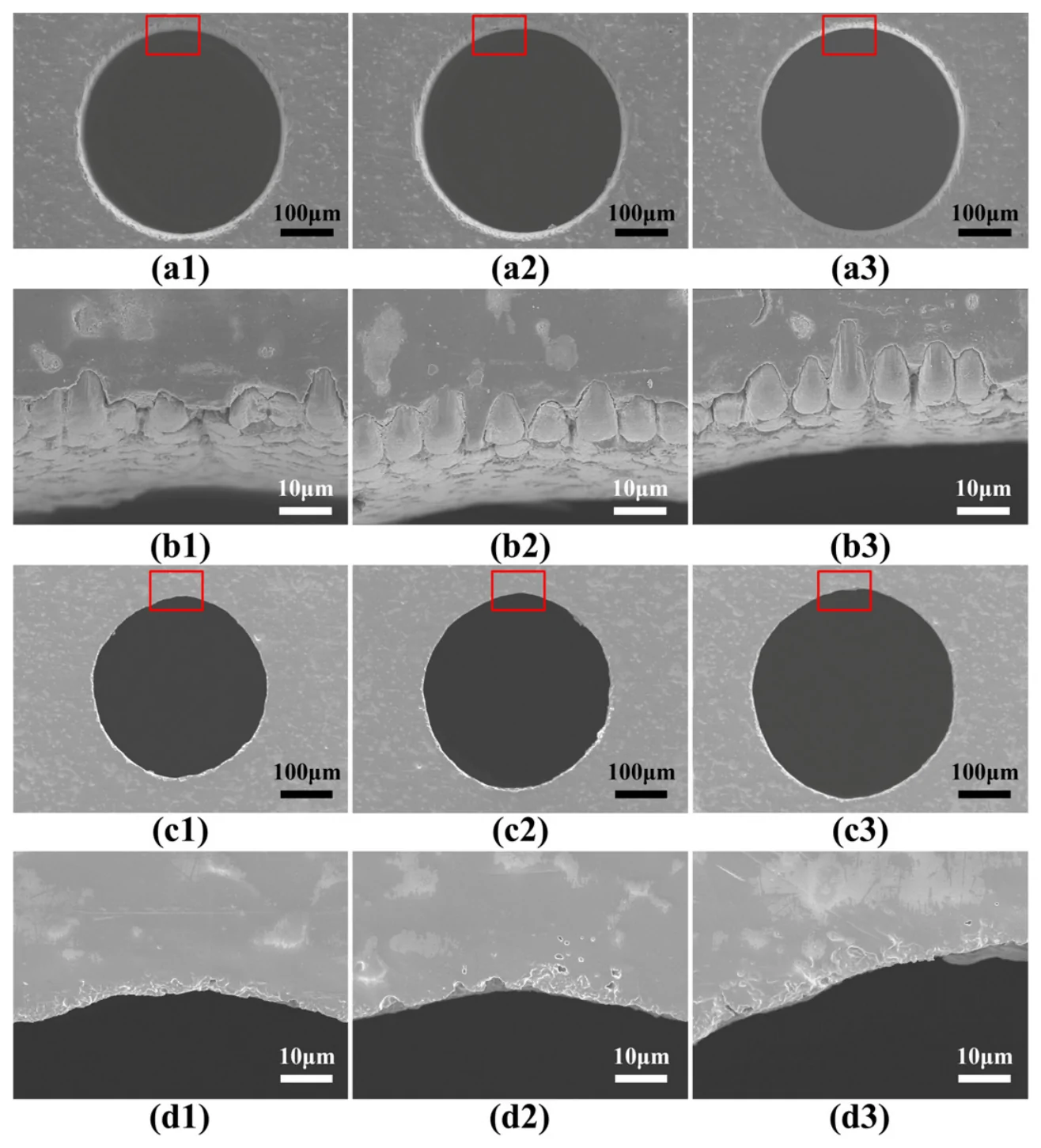
The entrance (**a1**–**a3**)–(**b1**–**b3**), and exit (**c1**–**c3**)–(**d1**–**d3**) after two-step processing with WD of 45 mm, 50 mm, and 55 mm, respectively. (**b1**–**b3**) and (**d1**–**d3**) are enlarged views of the edge regions indicated by the squares in (**a1**–**a3**) and (**c1**–**c3**), respectively. Here lasers repetition frequency is kept at 20 kHz.

**Figure 16:**
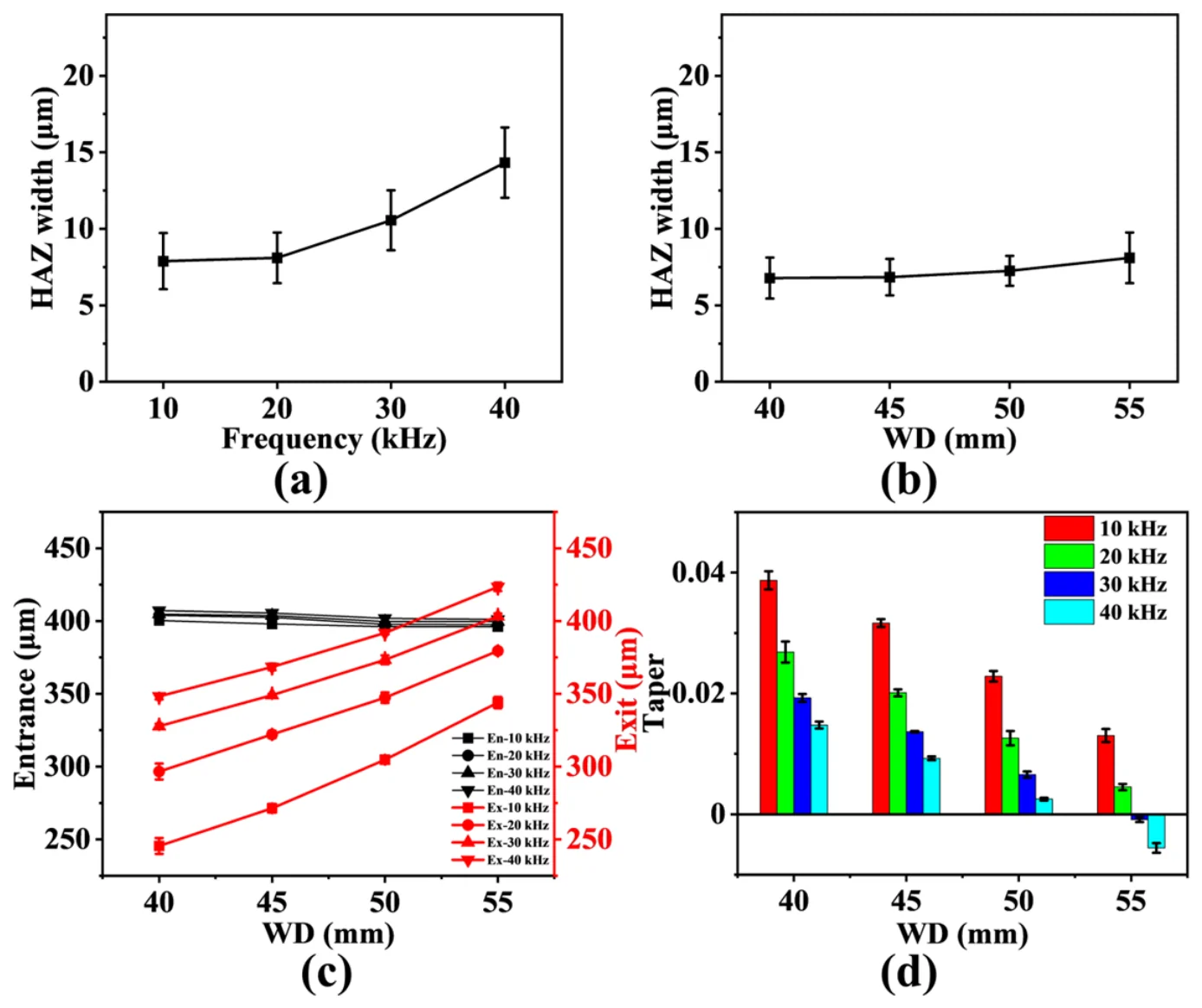
Dependence of HAZ width on pulse repetition frequency (**a**), and on WD (**b**). Dependence of entrance diameter and exit diameter (**c**), and taper (**d**), on both pulse repetition frequency and WD.

**Figure 17:**
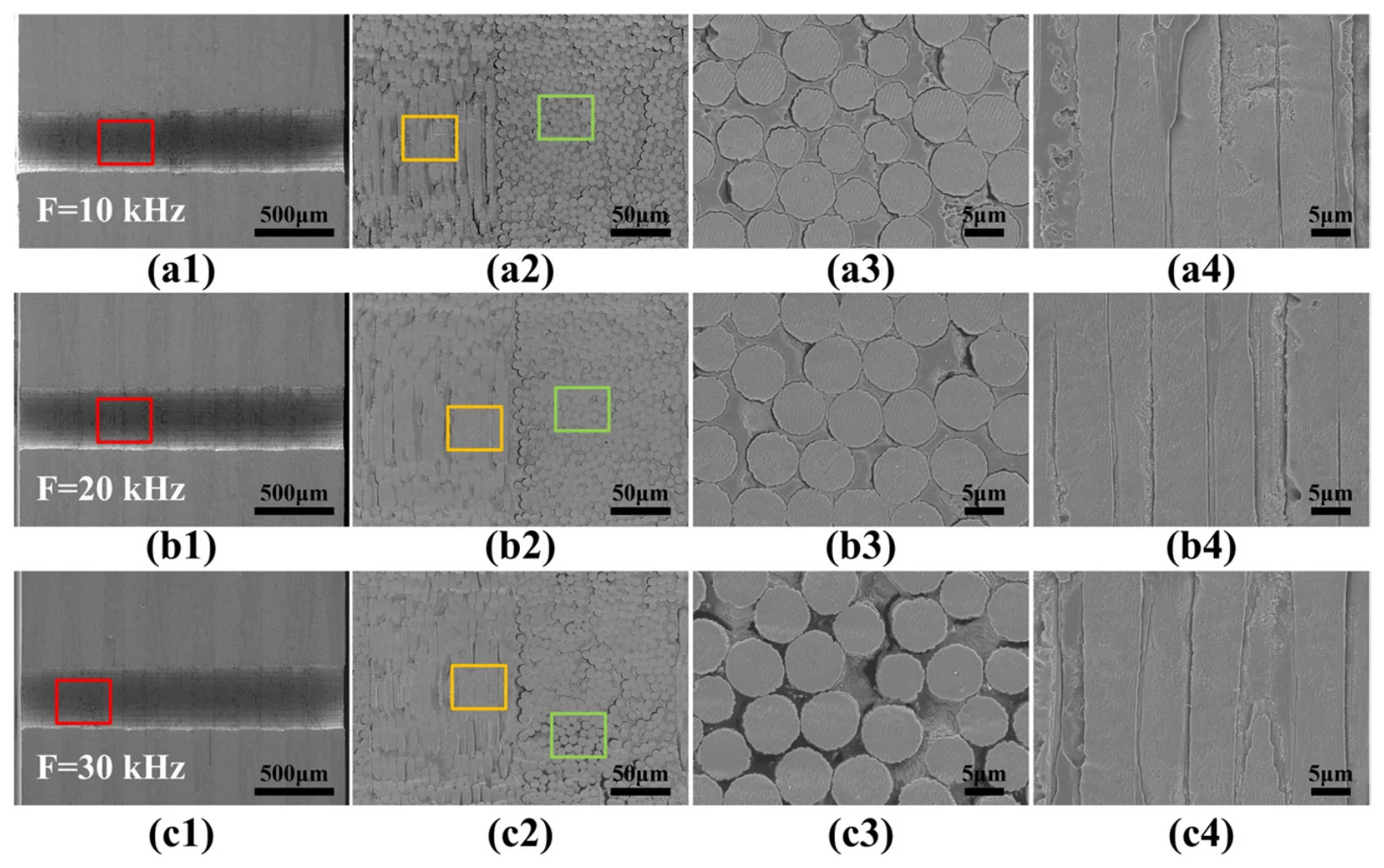
SEM images of the inner walls of micro-holes processed by lasers with different repetition frequencies. The WD was maintained at 55 mm. The pulse repetition frequencies for rows (**a**–**c**) were 10 kHz, 20 kHz, and 30 kHz, respectively. Column (**2**) shows the magnified views of the boxed regions in column (**1**). Columns (**3**) and (**4**) present further magnified views of the areas within the green and orange boxes in column (**2**), respectively.

**Figure 18:**
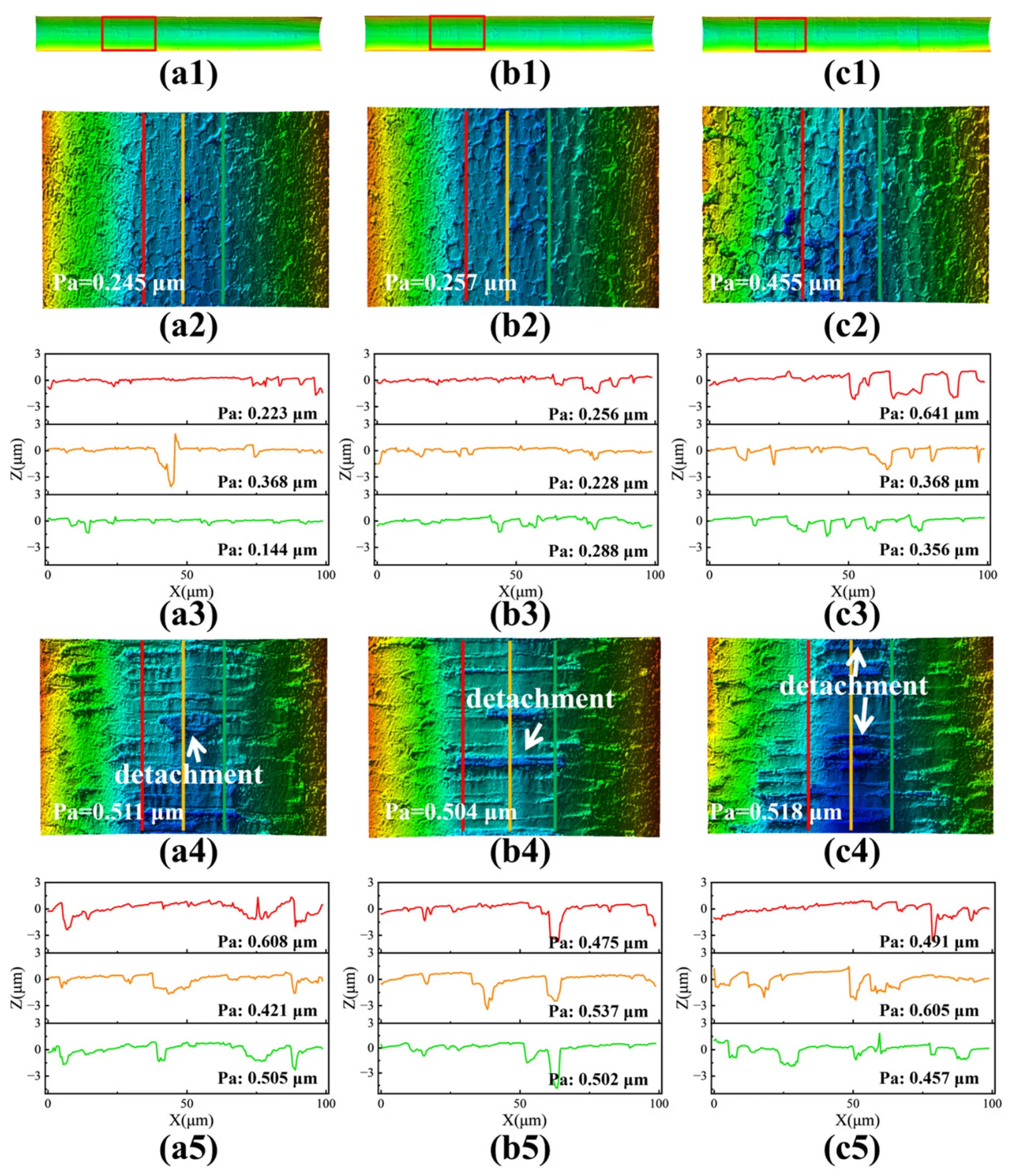
COM images and surface roughness analysis of the inner wall. (**a1**–**c1**) correspond to the overall morphology of the inner wall for pulse repetition frequencies of 10, 20 and 30 kHz, respectively. Rows (**2**,**3**) present the locally magnified COM images and roughness analysis for layers with vertically aligned fibers, while rows (**4**,**5**) are for layers with horizontally aligned fibers. The rainbow colors in the COM images (rows (**2**,**4**)) represent the relative surface height, ranging from −13.5 μm (purple) to 13.5 μm (red).

**Figure 19:**
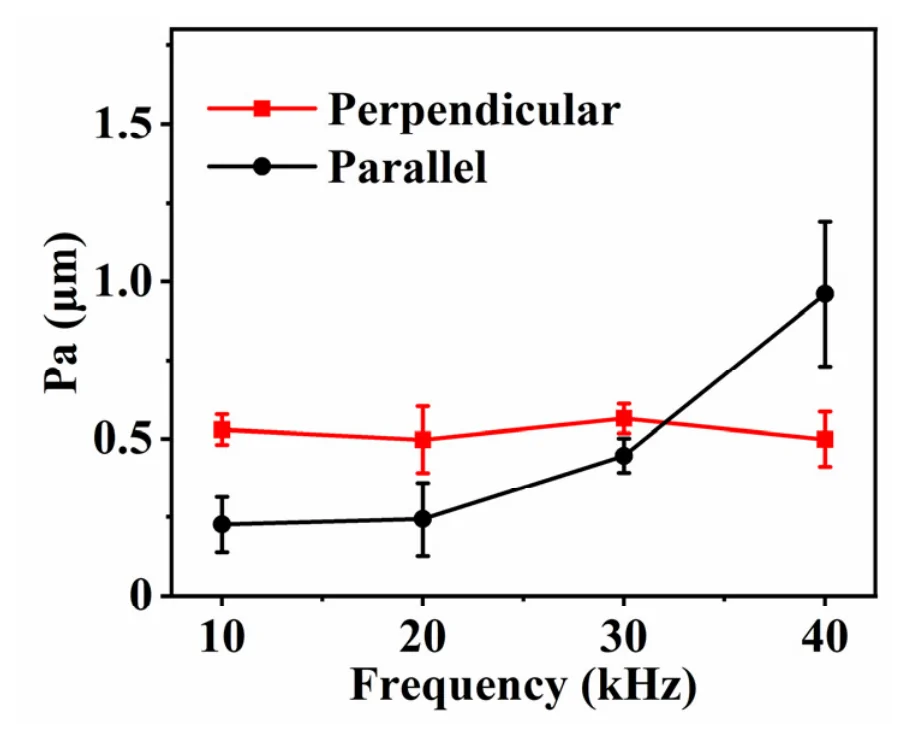
Roughness of the vertically and perpendicularly aligned fiber layers versus the pulse repetition frequency.

**Figure 20:**
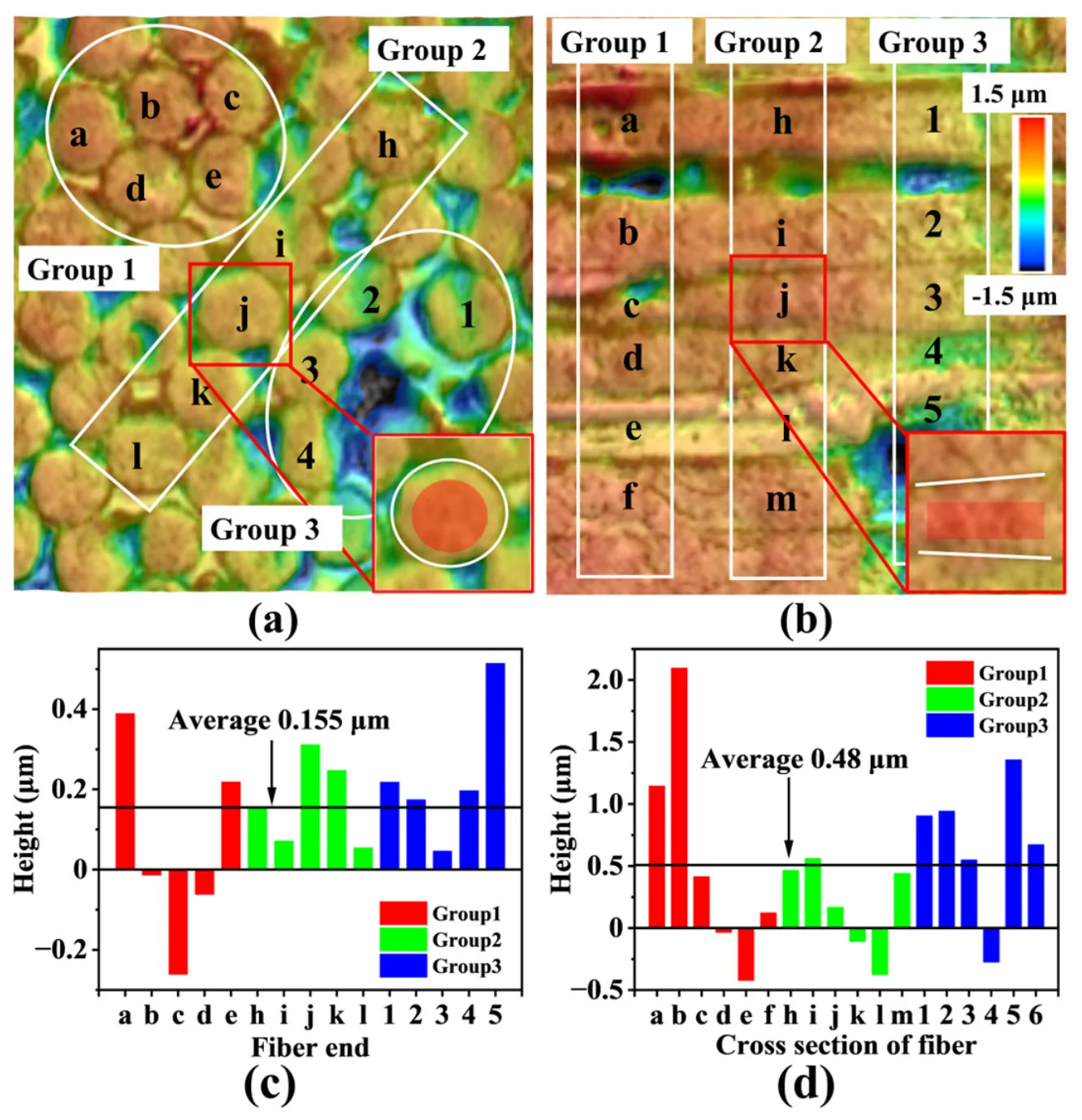
(**a**) COM images of fiber layers perpendicular to and (**b**) parallel to the bottom of the inner wall, processed with a laser at a repetition frequency of 20 kHz. The height scale bar is displayed on the right side of (**b**). (**c**,**d**) The statistical results of the height differences between the vertical fiber ends and the parallel fiber cross-sections relative to the adjacent resin, respectively.

**Figure 21:**
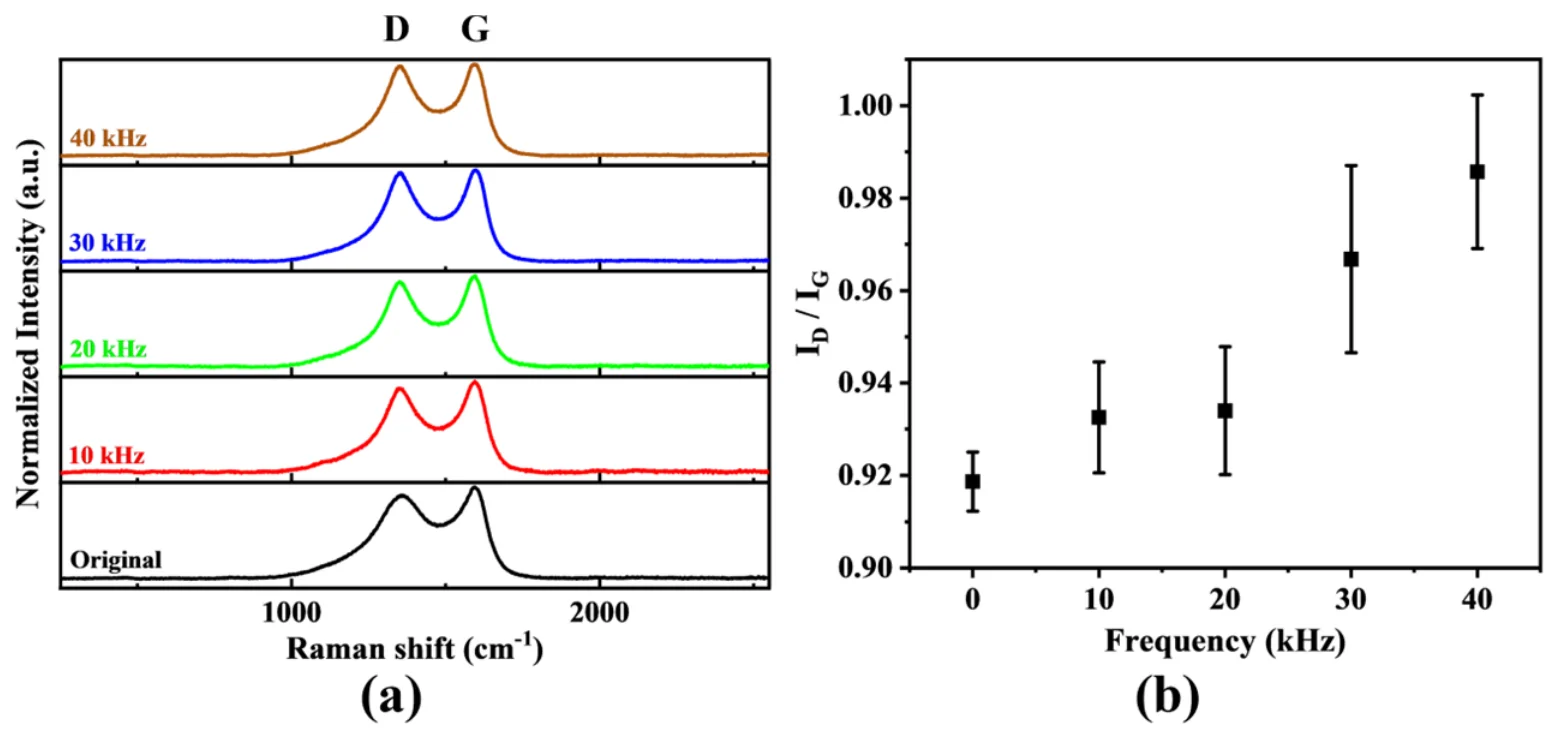
(**a**) Raman spectra of the carbon fiber center, and (**b**) *I_D_*/*I_G_* intensity ratio for different laser frequencies.

**Figure 22:**
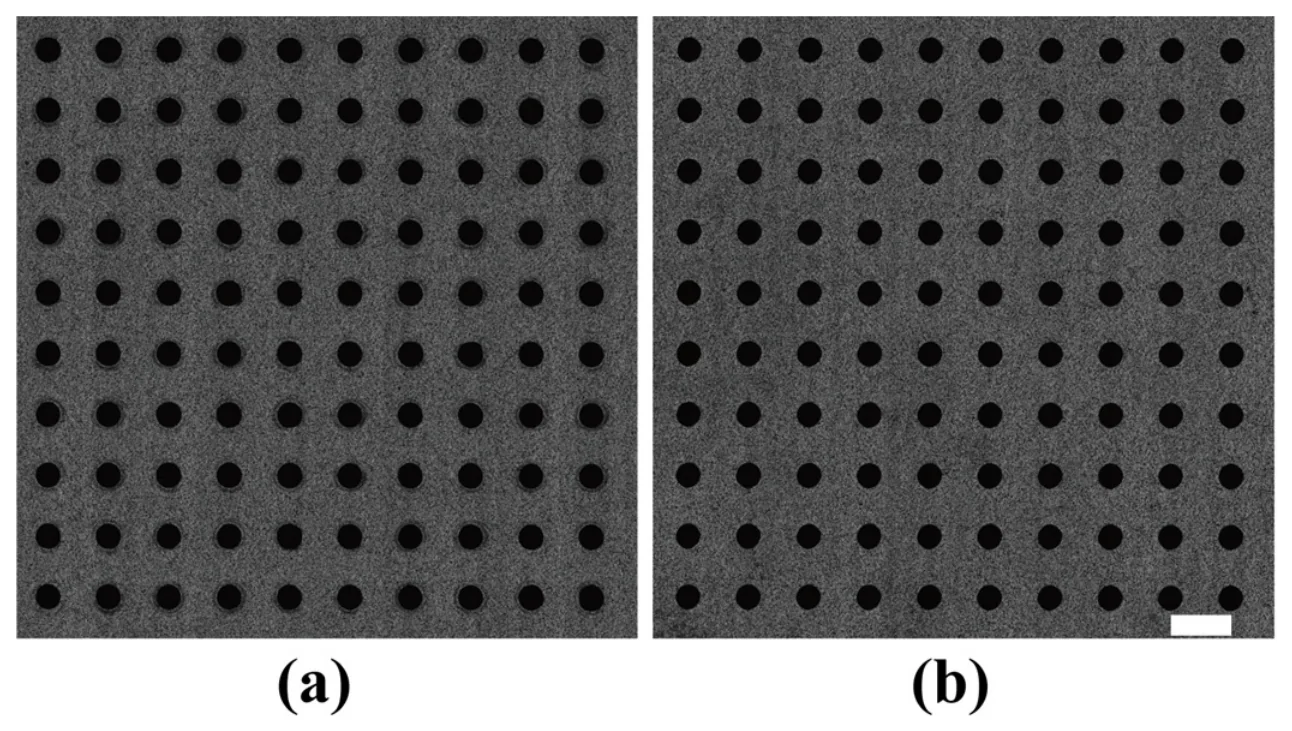
COM images of a 10 × 10 array of deep micro-holes: (**a**) entrance and (**b**) exit. The scale bar is 1 mm.

**Table 1:** The physical properties of the constituent materials in CFRP.

Property	Epoxy Resin	Carbon Fiber
Density (kg/m^3^)	1200	1850
Heat capacity (J/kg/K)	1884	710
Thermal conductivity (W/m/K)	0.1	50 (axial), 5 (radial)
Decomposition/vaporization temperature (K)	560–770	3900

**Table 2:** Laser parameters and processing conditions.

Parameters	Values	Units
Wavelength (λ)	1030	nm
Pulse duration (τ)	2	ps
Pulse energy (E)	90	μJ
Max repetition frequency (F)	1000	kHz
Focal length (f)	100	mm
Focused spot diameter	25	μm
Rotation speed of SSMH	2000	RPM
Number of spiral line variable radius turns	15	turn
Air pressure (p)	0.65	MPa

**Table 3:** AOI changing with WD.

WD (mm)	10	20	30	40	50
AOI (°)	0.16	−1.01	−1.48	−2.10	−3.37

**Table 4:** A comparative analysis of entrance/exit morphologies, and inner wall quality between this study and previous reports processed by ultrafast lasers.

Ref.	Diameter (mm)	Aspect Ratio	Taper (×10^−2^)	Drilling Speed (μm/s)	HAZ (μm)	Pa (μm)	Protrusion Height (μm)	I_D_/I_G_
This work	0.4	5	0.45	11.1	8.1	0.244 ^⊥^, 0.497 ″	0.155 ^⊥^, 0.48 ″	0.94 ^⊥^
[40]	0.21	1.43	4.15	4.3 *	15 *	×	×	×
[29]	11.29	0.89	×	8.5 *	<60	×	×	×
[24]	1	0.32	5.5	1.0 *	6.47 *	×	×	×
[30]	5	0.8	1.1 *	59.7 *	50	6.23 ^⊥^, 4.74 ″	×	×

^⊥^ The fiber perpendicular to the inner wall. ″ The fiber parallel to the inner wall. * The parameter was obtained by measuring or calculating the corresponding results from the cited references. × The corresponding information was not found in the cited literature.

## Data Availability

The original contributions presented in the study are included in the article; further inquiries can be directed to the corresponding authors.
